# A single nucleotide polymorphism in an R2R3 MYB transcription factor gene triggers the male sterility in soybean *ms6* (Ames1)

**DOI:** 10.1007/s00122-021-03920-0

**Published:** 2021-07-28

**Authors:** Junping Yu, Guolong Zhao, Wei Li, Ying Zhang, Peng Wang, Aigen Fu, Limei Zhao, Chunbao Zhang, Min Xu

**Affiliations:** 1 Key Laboratory of Biotechnology Shaanxi Province, College of Life Sciences, Chinese Education Ministry’s Key Laboratory of Western Resources and Modern Biotechnology, Northwest University, Xi’an, 710069 China; 2 Soybean Research Institute, National Engineering Research Center for Soybean, Jilin Academy of Agricultural Sciences, Changchun, 130033 China

## Abstract

**Key message:**

Identification and functional analysis of the male sterile gene *MS6* in *Glycine max*.

**Abstract:**

Soybean (*Glycine max* (L.) Merr*.*) is an important crop providing vegetable oil and protein. The male sterility-based hybrid breeding is a promising method for improving soybean yield to meet the globally growing demand. In this research, we identified a soybean genic male sterile locus, *MS6*, by combining the bulked segregant analysis sequencing method and the map-based cloning technology. *MS6*, highly expressed in anther, encodes an R2R3 MYB transcription factor (GmTDF1-1) that is homologous to Tapetal Development and Function 1, a key factor for anther development in *Arabidopsis* and rice. In male sterile *ms6* (Ames1), the mutant allele contains a missense mutation, leading to the 76th leucine substituted by histidine in the DNA binding domain of GmTDF1-1. The expression of soybean *MS6* under the control of the *AtTDF1* promoter could rescue the male sterility of *attdf1* but *ms6* could not. Additionally, *ms6* overexpression in wild-type *Arabidopsis* did not affect anther development. These results evidence that GmTDF1-1 is a functional TDF1 homolog and L76H disrupts its function. Notably, GmTDF1-1 shows 92% sequence identity with another soybean protein termed as GmTDF1-2, whose active expression also restored the fertility of *attdf1*. However, *GmTDF1-2* is constitutively expressed at a very low level in soybean, and therefore, not able to compensate for the *MS6* deficiency. Analysis of the TDF1-involved anther development regulatory pathway showed that expressions of the genes downstream of TDF1 are significantly suppressed in *ms6*, unveiling that GmTDF1-1 is a core transcription factor regulating soybean anther development.

**Supplementary Information:**

The online version contains supplementary material available at 10.1007/s00122-021-03920-0.

## Introduction

Soybean (*Glycine max* (L.) Merr*.*) is a major crop providing plant protein and oil in the food supply, but it has a relatively low yield (Palmer et al. 2001). Hybrid breeding technology, which significantly increases the yield of major crops such as rice and maize, shows a great potential in improving the yield of soybean as well (Kim and Zhang 2018; Palmer et al. 2001). For instance, HybSoy1, the first officially approved and commercially applicable hybrid soybean, can increase the yield by 20.8% (Zhao et al. 2004). In the hybrid breeding system, male sterile lines are indispensable for avoiding the time-consuming and tedious artificial emasculation.

There are two types of male sterility (MS), i.e., genic male sterility (GMS) controlled by nuclear *MS* genes and cytoplasmic male sterility (CMS) co-conditioned by mitochondrial *CMS* genes and nuclear fertility restorer genes (Guo and Liu 2012). Based on the stability of the MS phenotype, GMS could be further subdivided into two groups, the stable GMS and the environmental-sensitive GMS (EGMS). The hybrid breeding systems applied today include the CMS-based three-line system and the EGMS-based two-line system (Kim and Zhang 2018). However, for the CMS-based three-line system, the genetic resources of fertility restorer lines are limited. Some types of CMS cytoplasm even show detrimental effects on crop performance, such as elevating disease susceptibility in maize T-type CMS lines (Levings 1990). As to the EGMS two-line system, the sterility of EGMS lines could be reversed when the growth environment changes unexpectedly, endangering the hybrid production process (Chen et al. 2011a). Comparatively, stable GMS lines can overcome the limitations of EGMS and CMS lines due to the stable sterility and wide-range choices of restorer lines. However, lack of maintainer lines has been restricted the application of GMS lines for a long time. Until recently, the transgenic-based Seed Production Technology (SPT) system, which creates artificial maintainer lines by introducing a fertility restorer gene, a pollen killer gene and a selection marker gene into the GMS lines, makes GMS applicable to hybrid production (Perez-Prat and van Lookeren Campagne 2002; Weber et al. 2009).

So far, 13 GMS loci have been reported in soybean, which are distributed on seven chromosomes and function in conditioning anther development, including *ms1*-*ms9*, *msp*, *msMOS*, *mst-M* and *ms*_*NJ*_ (Yang et al. 2014; Zhao et al. 2019; Nie et al. 2019; Thu et al. 2019). Deficiency of these loci confers recessive sporophytic male-sterile phenotypes. Among these mutants, *ms6* displays a stable non-pollen phenotype (Skorupska and Palmer 1989; Ilarslan et al. 1999), making it an idea material for developing the SPT system in soybean. There are two independent, spontaneous *ms6* mutants maintained as heterozygotes in Soybean Genetic Type Collection as T295H (+ /*ms6* (Ames1)) (Skorupska and Palmer 1989) and T354H (+ */ms6* (Ames2)) (Ilarslan et al. 1999), respectively. Comparative microscopic studies on fertile and sterile offspring from T354H showed that the cytological abnormalities in *ms6* anther firstly appear at the microspore mother cell (MMC) stage on tapetal and parietal layers, which possess more vacuoles in cells. Then, the tapetum in *ms6* undergoes a premature programmed cell death and degrades entirely in the late microspore stage, while the parietal layer in *ms6* is enlarged during the later development stages (Ilarslan et al. 1999). The reproductive cells in *ms6* exhibit abnormalities at the end of meiosis, forming partially separated tetrads, which subsequently collapse in the late microspore stage when the fertile microspores are processing the first mitosis (Ilarslan et al. 1999). The similar phenomenon was observed during the microsporogenesis in *ms6* (Ames1) mutant from T295H (Skorupska and Palmer 1989).

Previous studies have revealed that the *ms6* mutation is closely linked to the flower-color gene *W1* (Skorupska and Palmer 1989; Palmer et al. 1998; Ilarslan et al. 1999), and located in a 3.7 Mb region on chromosome 13 (Chr13) between simple sequence repeat (SSR) markers Satt030 and Satt149 (Yang et al. 2014). In this study, via BSA-sequencing and map-based cloning experiments, we further narrowed down the genetic region of *ms6* (Ames1) and identified the corresponding mutation, which is a missense mutation in the gene *Glyma.13G066600* (designated as *MS6*). *MS6* encodes a homolog of Tapetal Development and Function 1 (TDF1), an R2R3 MYB transcription factor, which is critical for regulating tapetal layer degeneration in *Arabidopsis* and rice (Zhu et al. 2008; Cai et al. 2015). Accordingly, we named the protein encoded by *MS6* as GmTDF1-1. Notably, all the components constituting the TDF1-involved regulatory pathway in *Arabidopsis* and rice were also found in soybean, but each has multiple paralogs, in which the expressions of genes acting downstream of *TDF1* were strongly suppressed in *ms6* flowers. These indicate that the TDF1-related regulatory pathway is likely conserved but more complicated in soybean due to the recent whole-genome duplication events. Results from this study provide new insights into the regulatory network in soybean anther development, and make the *ms6* mutant a practicable material for the SPT system to facilitate hybrid seed production in soybean.

## Materials and methods

### Primers

Primers used in the present study were listed in Table S1.

### Plant materials and growth conditions

The *ms6* mutant used in this study was derived from T295H (PI 533601, + /*ms6* (Ames1)), which was achieved from the collection of the National Plant Germplasm System (NPGS) in the United States. Allele *ms6* (Ames1) is referred to as *ms6* hereafter. To narrow down the genetic region of the *ms6* mutation, a BC_5_F_2_ segregating population was developed by using T295H as *ms6* donor and a wild-type (WT) cultivar ‘JiuB’, from Jilin, China, as a recurrent male parent. Additionally, 40 male-fertile accessions with different geographic origins, including 21 improved cultivars, 4 landraces and 15 wild soybeans (*G. soja*), were used for evaluating the conservation of the nucleotide mutated in the *ms6* allele (Table S2). The mapping population was planted on the farm of Fanjiatun, Jilin in summer. For other studies, soybean plants were grown in pots (two plants per pot) outdoors in summer and in the greenhouse in winter at 28 °C with a photoperiod of 16 h light /8 h dark, in Xi’an, China.

*Arabidopsis thaliana* and *Nicotiana benthamiana* plants were grown in soil in the greenhouse at 22 °C with a photoperiod of 16 h light /8 h dark, in Xi’an, China. The *Arabidopsis* germplasms used in this study were WT Columbia (Col), heterozygous *attdf1* mutant (+ /*attdf1*) in Col background (obtained from Dr. Zhongnan Yang’s lab) and transgenic lines for the complementary and overexpression experiments. The *attdf1* mutation is caused by a single nucleotide polymorphism (SNP) that leads to a premature stop codon in *AT3G28470* (*AtTDF1*) and simultaneously creates an *Mse*I site (Zhu et al. 2008).

### Morphological and cytological analyses

For general morphological observation of *ms6* anthers, soybean flowers one day before blooming were collected from the fertile and sterile descendants of T295H (+ /*ms6*). Stamens were dissected and imaged under a stereo microscope Nikon SMZ25. Pollens were squeezed out, stained with 1% I_2_-KI solution and photographed under a light microscope Leica DM2500. For *Arabidopsis*, mature anthers before anthesis were collected from WT, homozygous *attdf1* mutant and various transgenic lines, stained with Alexander staining buffer (Peterson et al. 2010) and photographed under Leica DM2500.

For cytological analysis, flowers at the late tetrad and early pollen stages were collected from the fertile and sterile descendants of T295H (+ /*ms6*) and immediately immersed into the FAA fixation solution. After dehydration, flower samples were imbedded into resin with Technovit H7100-GMA kit (Heraeus Kulzer, Germany) following the manufacturer’s instruction and sliced into 2-μm transverse sections with Leica RM2265. Sections on slides were stained with 0.5% toluidine blue staining buffer and imaged under Leica DM2500 after sealed.

### DNA extraction, BSA-sequencing (BSA-seq) and fine mapping analyses

Genomic DNA samples were extracted from young leaves with the Nuclean Plant Genomic DNA Kit (CWBIO, China) for regular PCR analysis and BSA-seq experiment. For BSA-seq analysis, two bulks were constructed from the BC_5_F_2_ mapping population. One was composed of 20 homozygous WT plants and the other was composed of 20 homozygous *ms6* plants. Genomic DNA isolated from each bulk was fractioned to build a 350-bp pair-end (PE) sequencing library and sequenced on Illumina NovaSeq 6000 PE150 platform in Novogene Company (China). SNPs and InDels (insertions-deletions) of each bulk were annotated using the Wm82.a2.v1 genome as the reference. The SNP-index of each bulk and the difference between the SNP-index of two bulks, ∆(SNP-index), were calculated as previously described (Takagi et al. 2013). Then, ∆(SNP-index) values were plotted against their genome positions to identify the region associated with *ms6*.

Polymorphic SSR markers in the genetic window of *ms6* identified by BSA-seq were further used to screen the *ms6* individuals in the BC_5_F_2_ population via canonical PCR. The fragments amplified with SSR primers were resolved on 8% polyacrylamide gel in 1xTAE buffer by electrophoresis and visualized by the silver staining method (Bassam et al. 1991). The genetic map was constructed from the data with MAPMAKER 3.0 (Lander et al. 1987).

### Cleaved amplified polymorphic sequence (CAPS) analysis

CAPS analysis was performed to genotype the *MS6* locus in soybean and the *AtTDF1* locus in *Arabidopsis*. The genomic regions of *MS6* or *AtTDF1* spanning the SNP site were amplified from testing samples by PCR with gene specific primers. Subsequently, five microliters of PCR products were digested with *Mse*I according to the manufacturer’s instruction, and the band patterns were analyzed on 4% agarose gel.

### Bioinformatics and phylogenetic analyses

Conserved structural domain in the GmTDF1-1 polypeptide was predicted by the simple modular architecture research tool (SMART) (http://smart.embl-heidelberg.de/), showing GmTDF1-1 contained a typical R2R3 MYB DNA-binding domain. The amino acid sequences of the R2 region of GmTDF1-1 and 23 well-characterized MYB proteins in *Arabidopsis* (Dubos et al. 2010) were aligned with Bioedit software (Tom Hall, Ibis Biosciences, U.S.) to access the conservation of the R2 region of GmTDF1-1. The proteins with highest homology to GmTDF1-1 in *G. max* (GmTDF1-2), *A. thaliana* (AtTDF1) and *Oryza sativa* (OsTDF1) were identified in NCBI Database with BLASTP, and the conservation between these three proteins and GmTDF1-1 was evaluated with Bioedit software as well.

The amino acid sequences of 17 proteins were subjected to phylogenetic analysis, including GmTDF1-1, GmTDF1-2, AtTDF1, OsTDF1 and the putative TDF1 orthologs in 13 additional representative species from different land plant evolutionary lineages, including three dicot (*Medicago truncatula*, *Vitis vinifera* and *Solanum lycopersicum*), five monocot (*Zea mays*, *Sorghum bicolor*, *Ananas comosus*, *Musa acuminate* and *Zostera marina*), one basal angiosperm (*Amborella trichopoda*), two gymnosperm (*Ginkgo biloba* and *Pinus taeda*), one lycophyte (*Selaginella moellendorffii*) and one moss (*Physcomitrella patens*) species. The sequences of the putative TDF1 orthologs were retrieved from NCBI with BLASTP by using AtTDF1 (NP 189,488.1) and OsTDF1 (XP 015,630,216.1) as query peptides, and the one with the highest bit-score in each species was selected. AtMYB80 (NP 200,422.1) and OsMYB80 (XP 015,635,420.1) were used as outgroup sequences. All the protein sequences were aligned using the ClustalW2 with default parameters, and the phylogenetic tree was constructed by the neighbor-joining method with 1000 bootstrap replicates using MEGA 6 (Tamura et al. 2013).

### RNA extraction, RT-PCR and qRT-PCR analyses

DNA-free total RNA was extracted from desired tissues with RNAprep Pure Plant Kit (Tiangen, China). One microgram of RNA was reverse transcribed to cDNA using PrimeScript™ II 1st Strand cDNA Synthesis Kit (Takara, Japan). RT-PCR was conducted with rTaq DNA polymerase (Takara, Japan) for analyzing gene expression or with 2 × PrimeSTAR Max Premix (Takara, Japan) for cloning purposes following the manufacturer’s instructions. For quantitative RT-PCR, one microliter of 1:10 diluted cDNA sample was used as a template, and the qRT-PCR reaction was conducted as previously described (Zhang et al. 2018a). The relative expression levels of *MS6* and *GmTDF1-2* in various WT tissues were calculated with the 2^−∆Ct^ method (Livak and Schmittgen 2001). The fold-changes of target genes in young flowers (2.5–3.5 mm in length) of *ms6* compared to those of WT were calculated with the 2^−∆∆Ct^ method (Livak and Schmittgen 2001). All data were normalized against the expression level of *GmActin11* (*Glyma.18G290800*). Three biological replicates, each with three-technique replicates, were performed for each sample.

### Subcellular localization analysis

The coding sequences (CDS) for proteins GmTDF1-1 and GmTDF1-1^L76H^ (the mutant protein encoded by the *ms6* allele) without stop codon were amplified by RT-PCR from WT and *ms6* flowers, respectively, and cloned into the *Xba*I site upstream of the *GFP* gene in the binary vector pLM-35S-GFP to create pLM-35S-GmTDF1-1-GFP and pLM-35S-GmTDF1-1^L76H^-GFP, respectively. Vectors were transformed into *Agrobacterium tumefaciens* strain GV3101 and infiltrated into 4-week-old *N. benthamiana* leaves. GFP signals in leaves were observed and imaged at 48 h post infiltration under an Olympus Fluoview FV1000 confocal laser scanning microscope.

### Transactivation activity assay in yeast

The CDSs for GmTDF1-1 and its DNA binding domain (amino acids 1–191, GmTDF1-1^DBD^) were amplified by RT-PCR from WT flowers; the CDS for GmTDF1-1^L76H^ was amplified by RT-PCR from *ms6* flowers. The obtained fragments were cloned into the pGBKT7 vectors downstream of the CDS for GAL4-BD, respectively, to generate the vectors of pGBKT7-GmTDF1-1, pGBKT7-GmTDF1-1^DBD^ and pGBKT7-GmTDF1-1^L76H^, which were subsequently transformed into *Saccharomyces cerevisiae* strain AH109 via one-step transformation method (Chen et al. 1992). After selected on the synthetic dropout medium lack of trypsin (SD/-Trp), the positive colonies were diluted into the same concentration with autoclaved ddH_2_O. Then, five microliters of cell suspensions were dropped on the selective medium SD/-Trp/-His and grown for 3–4 days under 30 °C to evaluate the activation activities of target proteins.

### Complementary and overexpression analyses

For complementary analysis, the 817-bp *AtTDF1* promoter reported previously by Zhu et al. (2008) was cloned into pCAMBIA1301 via *Kpn*I and *Xba*I to get the pCAMBIA1301-*AtTDF1p* vector. The CDSs of *MS6*, *ms6*, *AtTDF1*, *AtTDF1*^*L46H*^ and *GmTDF1-2* were cloned into pCAMBIA1301*-AtTDF1p* downstream the *AtTDF1* promoter through *Xba*I and *BstE*II to acquire pCAMBIA1301-*AtTDF1p*-*MS6*, pCAMBIA1301-*AtTDF1p*-*ms6*, pCAMBIA1301-*AtTDF1p*-*AtTDF1*, pCAMBIA1301-*AtTDF1p*-*AtTDF1*^*L46H*^ and pCAMBIA1301-*AtTDF1p*-*GmTDF1-2*, respectively. Here, *AtTDF1*^*L46H*^ is a variant of *AtTDF1* CDS generated by site-directed mutagenesis via overlap extension PCR (Ho et al. 1989), and it encodes a mutant form of AtTDF1 (AtTDF1^L46H^) corresponding to GmTDF1-1^L76H^.

*A. tumefaciens* strain GV3101 carrying these vectors were used to transform *Arabidopsis attdf1* heterozygous plants by the floral-dip method (Clough and Bent 1998). T1 transformants were screened on 1/2 MS agar plates supplemented with 1% sucrose and 20 mg/L hygromycin. The hygromycin-resistant T1 plants were transplanted into soil for further growth after they were verified for the presence of the transgene by PCR. The native *AtTDF1* locus in each T1 transgenic plant was genotyped by CAPS analysis. Transgenic plants in homozygous *attdf1* background were further scored for fertility.

For overexpression analysis, the CDS of the *ms6* allele was cloned into the PLM-35S vector via *Xba*I and *Eco*RI site to generate PLM-35S-*ms6*, which was transformed into WT *Arabidopsis* (Col) via *Agrobacterium* as described above. T1 transformants were selected by Basta resistance, confirmed by PCR analysis and further scored for anther fertility. The expression level of *ms6* in T1 transgenic plants was examined by RT-PCR with *AtActin* as a reference gene.

## Results

### Phenotypic characterization of the *ms6* mutant

**Fig. 1:**
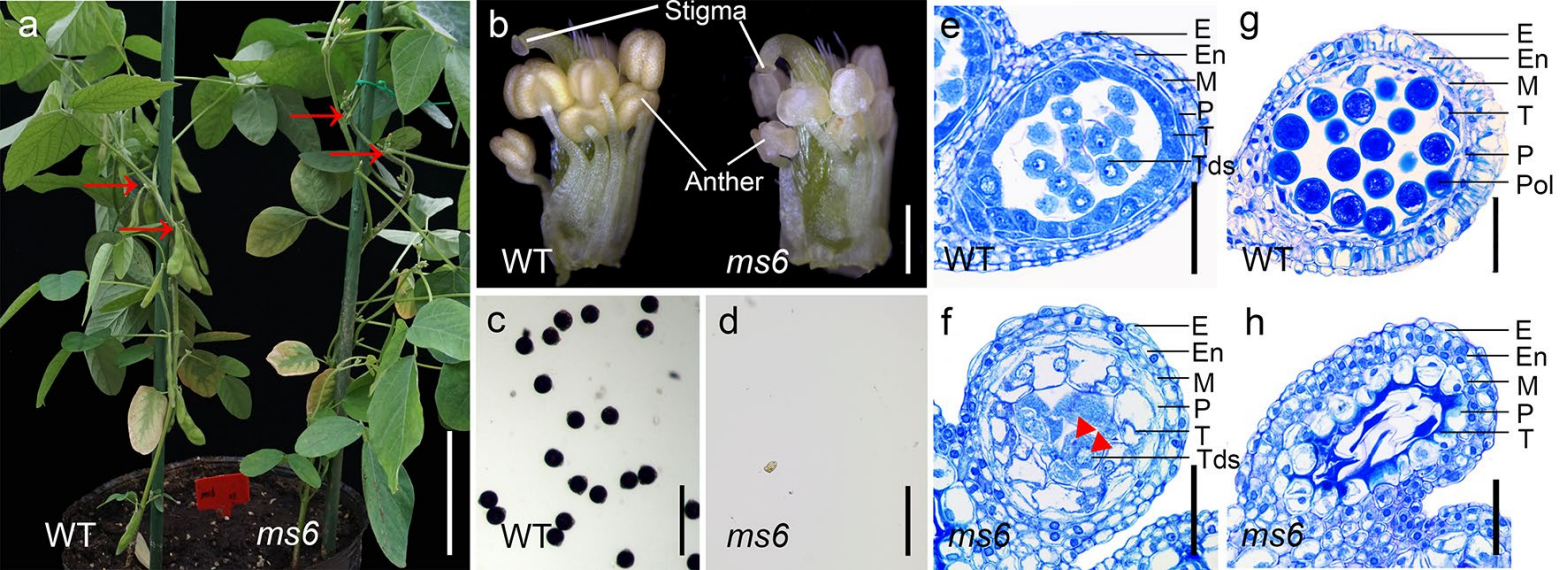
Phenotypic characterization of *ms6* in soybean **a** Wild-type (WT) and *ms6* plants at R3 stage of WT. Compared with WT, *ms6* fails to produce elongated pods at nodes (red arrow). Scale bar = 10 cm. **b** WT and *ms6* flowers with petals and sepals removed. Scale bar = 0.5 mm. **c**, **d** I_2_-KI staining of pollens squeezed out from WT and *ms6* anthers. Scale bar = 50 μm. **e**–**h** Semi-thin sections of WT and *ms6* anther lobes at the late tetrad stage **e**, **f** and the early pollen stage **g**, **h**. Scale bar = 50 μm. Red triangles indicate the multi-nuclei in tetrads. E, Epidermis; En, Endodermis; M, Middle layer; P, Parietal layer; T, Tapetal layer; Tds, Tetrads; Pol, Pollen (color figure online)

The offsprings of heterozygous *ms6* plants (T295H) were planted in the greenhouse. During the vegetative stage, all plants grew well just like WT plants, while at the reproductive stage about a quarter of plants were male-sterile and failed to develop pods, indicating they were *ms6* homozygotes (Fig. 1a). The anthers of *ms6* plants were whitish and shrunken compared to those of WT plants (Fig. 1b). Moreover, pollen grains released from WT anthers were round and stained dark blue by I_2_-KI solution (Fig. 1c), but no pollen grains were produced in *ms6* anthers (Fig. 1d). These results are consistent with the previous report (Skorupska and Palmer 1989).

The flowers of WT and *ms6* at the late tetrad and early pollen stages were cross-sectioned and observed under light microscopy. At the late tetrad stage, WT anther wall was composed of five layers, including epidermis, endothecium, middle layer, parietal layer and tapetum from outside to inside, in which the tapetum cells exhibited highly condensed cytoplasm. Meanwhile, callose surrounding the tetrads in anther locule started to degenerate (Fig. 1e). Anthers at the same development stage in *Arabidopsis* and rice show similar cytological features except that their anther walls do not have a parietal layer (Sanders et al. 1999; Zhang et al. 2011). On the other hand, *ms6* anthers at this stage had radically enlarged and highly vacuolated parietal and tapetal layers; in the locule, partially or non-separated irregular microspores with multiple nuclei were encased in callose, indicating that cytokinesis II of meiocytes was abnormal (Fig. 1f). By the time of the early pollen stage, enlarged pollens with thick wall were observed in the locule of WT anthers, and the anther wall was composed of an epidermis, an enlarged endothecium and a narrow parietal layer attached by tapetum remnants (Fig. 1g). Comparatively, in *ms6* anthers, tapetum cells completely degraded and pollens collapsed while the parietal layer became abnormally vacuolated and swollen (Fig. 1h). These observations are similar to those of *ms6* (Ames2), except that the tapetal layer of *ms6* (Ames2) degenerated more rapidly. It almost completely degraded at the late tetrad stage (Ilarslan et al. 1999).

### An SNP in *Glyma*.*13G066600* is likely responsible for the male sterility in *ms6*

**Fig. 2:**
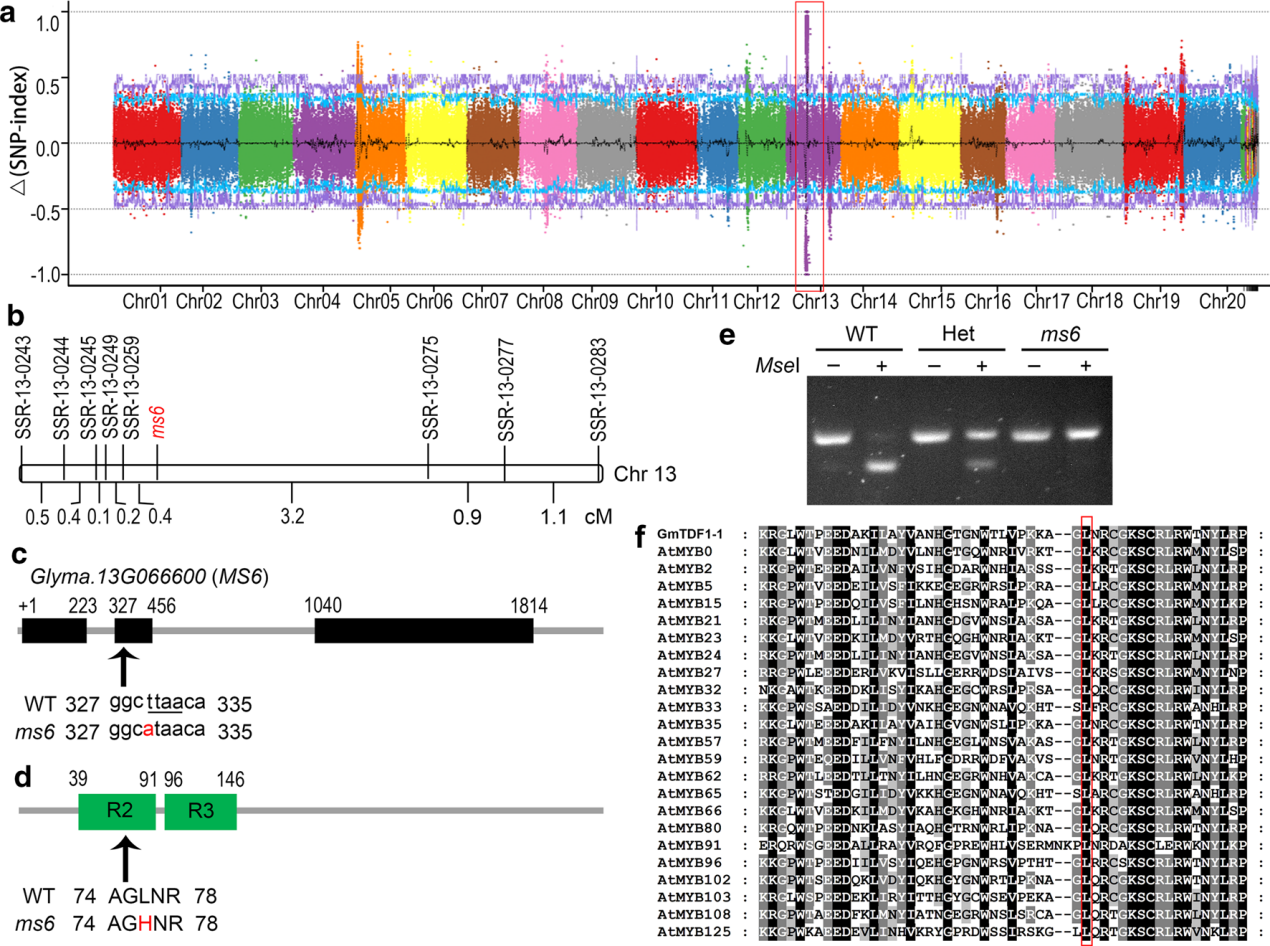
*MS6* encodes an R2R3 MYB transcription factor **a** Manhattan plot of the SNP-index differences, ∆(SNP-index), between WT and *ms6* bulks constructed with a BC_5_F_2_ mapping population derived from *ms6* x ‘JiuB’. The red box highlights the region co-segregated with *ms6*. **b** Genetic linkage map of *ms6*. Genetic distances between two adjacent loci are indicated by centimorgans (cM). Chr 13, chromosome 13. **c** The *MS6* gene structure and the mutated site in *ms6*. Black boxes represent the exons of *MS6*; arrow indicates the SNP location; nucleotide in red is the mutated site in *ms6*; underlined sequence is the *Mse*I restriction site. **d** The GmTDF1-1 protein structure. Green blocks indicate the R2R3 MYB DNA-binding domain; arrow and amino acid in red indicate the amino acid substitution site in *ms6*. **e** Genotyping the plants with the CAPS marker based on the SNP between WT and *ms6*. The *Mse*I-undigested (−) or digested ( +) PCR products are segregated on 4% agarose gel. WT, homozygous wild-type plant; Het, heterozygous (+ */ms6*) plant; *ms6*, homozygous *ms6* mutant. **f** Multiple sequence alignment of the R2 region from different MYB proteins. The residue corresponding to L76 in GmTDF1-1 (indicated by the red box) is conserved (color figure online)

Previously, the *ms6* mutation was mapped within a 3.72 Mb region on Chr13 between SSR markers Satt149 (Chr13:13,134,055 bp) and Satt030 (Chr13:16,855,019 bp) (Yang et al. 2014). To narrow down the region, WT and *ms6* bulks, constructed from a BC_5_F_2_ mapping population originated from the cross of the *ms6* mutant and ‘JiuB’, were subjected to the BSA-seq analysis. The ∆(SNP-index) plot showed that the *ms6* mutation was associated with a 1.5 Mb region on Chr13 (Chr13:15,853,267–17,349,424 bp) (Fig. 2a), consisting to the previously reported interval (Yang et al. 2014). In addition, 214 SNP and 35 InDel variations were identified between two bulks in this region.

Subsequently, a fine mapping was conducted with 328 individual plants in the BC_5_F_2_ mapping population with 9 polymorphic SSR markers identified in the 1.5 Mb interval, including BARCSOYSSR-13–0243, BARCSOYSSR-13–0244, BARCSOYSSR-13–0245, BARCSOYSSR-13–0249, BARCSOYSSR-13–0257, BARCSOYSSR-13–0259, BARCSOYSSR-13–0275, BARCSOYSSR-13–0277 and BARCSOYSSR-13–0283 (Fig. 2b; Fig. S1). Finally, the *ms6* mutation was restricted to a 255 kb region (Chr13:16,428,596–16,683,664 bp) between BARCSOYSSR-13–0259 and BARCSOYSSR-13–0275 (Fig. 2b), which harbors 23 annotated genes. Among these genes, only *Glyma.13G066600* showed a variation in the CDS region between WT and *ms6* bulks according to the BSA-Seq results. It is a T-to-A SNP at position 330 in exon 2 of *Glyma.13G066600*, which destroys an *Mse*I restriction site (Fig. 2c). Therefore, we verified this mutation by CAPS assay. A 126-bp region covering the SNP site was amplified by PCR from homozygous WT (+ / +), heterozygotes (+ /*ms6*) and homozygous *ms6* (*ms6*/*ms6*), and the PCR products were subsequently digested with *Mse*I. As expected, the WT amplicon was cleaved to a 99-bp band and a 27-bp band (invisible on the agarose gel); the *ms6* amplicon only showed a 126-bp band because it could not be digested by *Mse*I; the heterozygous amplicon had a 99-bp cut band and a 126-bp uncut band (Fig. 2e). Then, we further examined this position in 40 male-fertile wild, landrace and improved soybean accessions by the same CAPS assay, and they all exhibited the WT band pattern (Fig. S2, Table S2), indicating the position 330 of *Glyma.13G066600* is highly conserved.

The SMART analysis indicated the protein encoded by *Glyma.13G066600* is a transcription factor containing a typical R2R3 MYB DNA-binding domain at the *N*-terminal region (Fig. 2d). In the *ms6* mutant, the SNP in *Glyma.13G066600* leads to a L76H substitution (leucine at residue 76 substituted by histidine) in the polypeptide (Fig. 2d). Sequence comparison revealed L76 is well conserved in the R2 region of MYB proteins (Fig. 2f), implying it may be critical for the function of MYB proteins. The BLASTP analysis showed that *Glyma.13G066600* encoded a putative homolog of TDF1, a key transcription factor regulating tapetum development and function (Zhu et al. 2008; Cai et al. 2015). The protein encoded by *Glyma.13G066600* shares the highest sequence homology to AtTDF1 (44.9% identity and 64.6% similarity) in *Arabidopsis* and OsTDF1 (35.0% identity and 55.8% similarity) in rice (Fig. S3). Moreover, the null mutants of *tdf1* in *Arabidopsis* and rice both promote vacuolization in tapetal cells and suppress the degradation of callose surrounding the tetrads, resulting in squeezed microspores and no-pollen phenotype (Zhu et al. 2008; Cai et al. 2015), which are similar to the phenotypes of *ms6*. These results together suggest that the T-to-A SNP in the CDS of *Glyma.13G066600* is responsible for the male sterility of soybean *ms6*, and therefore we termed *Glyma.13G066600* as *MS6*.

### *MS6* encodes a homolog of TDF1, an R2R3 MYB transcription factor only present in angiosperm

The above results indicated that the protein encoded by *MS6* was likely a TDF1 homolog and named as GmTDF1-1 accordingly, although its sequence similarities shared with AtTDF1 and OsTDF1 were relatively low (Fig. S3). The relatively low homology was attributed to the highly diverged C-terminal region, which is a characteristic of MYB transcription factors (Jin and Martin 1999). Additionally, GmTDF1-1 exhibited a strong sequence homology (92% identity) to another soybean protein, which is encoded by *Glyma.19G017900* and designated as GmTDF1-2 (Fig. S3). To verify the relationship between GmTDF1-1, GmTDF1-2 and TDF1, we conducted a phylogenetic analysis of GmTDF1-1, GmTDF1-2, AtTDF1, OsTDF1 and the putative homologs of TDF1 in other land plant species from different evolutionary lineages. AtMYB80 and OsMYB80 were used as outgroup sequences since MYB80 is the closest homologous protein of TDF1 (also known as MYB35) (Dubos et al. 2010).

**Fig. 3:**
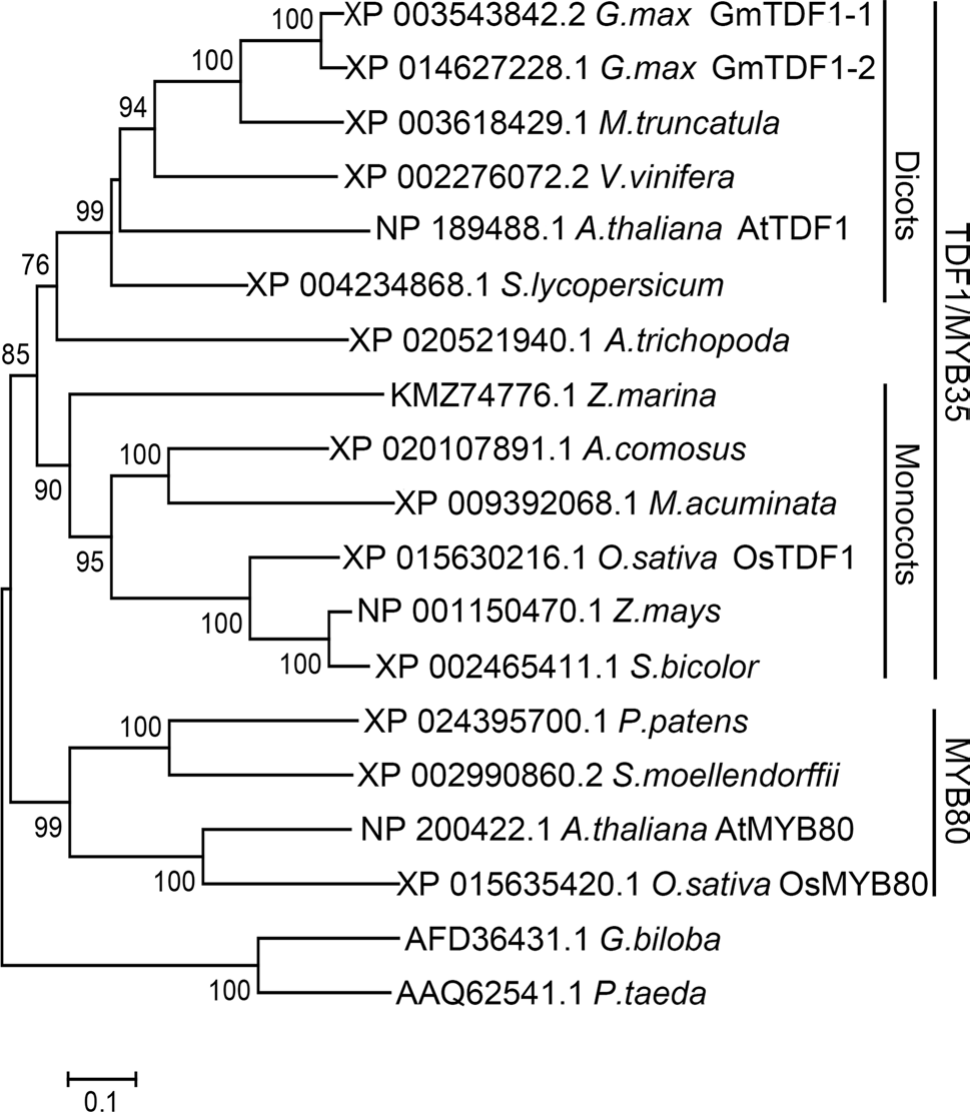
Phylogenetic tree of GmTDF1-1 and TDF1 homologs from different plant species. The unrooted neighbor-joining phylogenetic tree based on GmTDF1-1 and TDF1 homologs is created by MEGA6. *A. thaliana* (*Arabidopsis thaliana*), *A. trichopoda* (*Amborella trichopoda*), *A. comosus* (*Ananas comosus*), *G. biloba* (*Ginkgo biloba*), *G. max* (*Glycine max*), *M. acuminata* (*Musa acuminata*), *M. truncatula* (*Medicago truncatula*), *O. sativa* (*Oryza sativa*), *P. taeda* (*Pinus taeda*), *P. patens* (*Physcomitrella patens*), *S. bicolor* (*Sorghum bicolor*), *S. lycopersicum* (*Solanum lycopersicum*), *S. moellendorffii* (*Selaginella moellendorffii*), *V. vinifera* (*Vitis vinifera*), *Z. mays* (*Zea mays*) and *Z. marina* (*Zostera marina*). Bootstrap values are indicated on the branches

As a result, these proteins are clustered into three groups (Fig. 3). In particular, AtTDF1, OsTDF1 and their putative homologs in other angiosperm species including GmTDF1-1 and GmTDF1-2 are clustered into a monophyletic group with two sub-branches. Specifically, AtTDF1 and all the TDF1 putative homologs in dicots are grouped in the same sub-branch with the one in the basal angiosperm *A. trichopoda*. OsTDF1 and all the TDF1 putative homologs in monocots including the basal or near basal monocots *A. comosus* and *Z. marina* are grouped in the other sub-branch (Fig. 3). Nevertheless, the TDF1 homologous proteins with the highest bit-score in lycophyte *S. moellendorffii* and moss *P. patens* form a second cluster with MYB80, and those in gymnosperm species, *G. biloba* and *P. taeda*, form the third cluster (Fig. 3). These data suggest that TDF1 is only present in angiosperm and has diverged at a very early stage in angiosperm evolution before monocots and dicots were differentiated. Moreover, the fact that GmTDF1-1 and GmTDF1-2 are placed together in the sub-branch of dicot TDF1s demonstrates that they are TDF1 homologs in soybean and their genes have arisen from a recent duplication event (Fig. 3).

### L76H mutation abolished the ability of GmTDF1-1 to complement the male sterility of *Arabidopsis attdf1* mutant

The function of TDF1 was likely conserved in angiosperm. Firstly, phylogenetic results showed that TDF1 homologs in angiosperm were clustered into a monophyletic group (Fig. 3). Secondly, depletion of TDF1 in *Arabidopsis* and rice caused similar detrimental effects on tapetum development (Zhu et al. 2008; Cai et al. 2015). Thirdly, expression of OsTDF1 driven by the native promoter *AtTDF1p* could rescue the male sterility of *attdf1* (Cai et al. 2015). Therefore, to verify that GmTDF1-1 is a functional TDF1 critical for anther development and L76H mutation in *ms6* disrupts the protein function, we conducted a complementary experiment by transferring the CDSs of *AtTDF1*, *MS6*, *ms6* and *GmTDF1-2* driven by *AtTDF1p* into *attdf1*.

**Fig. 4:**
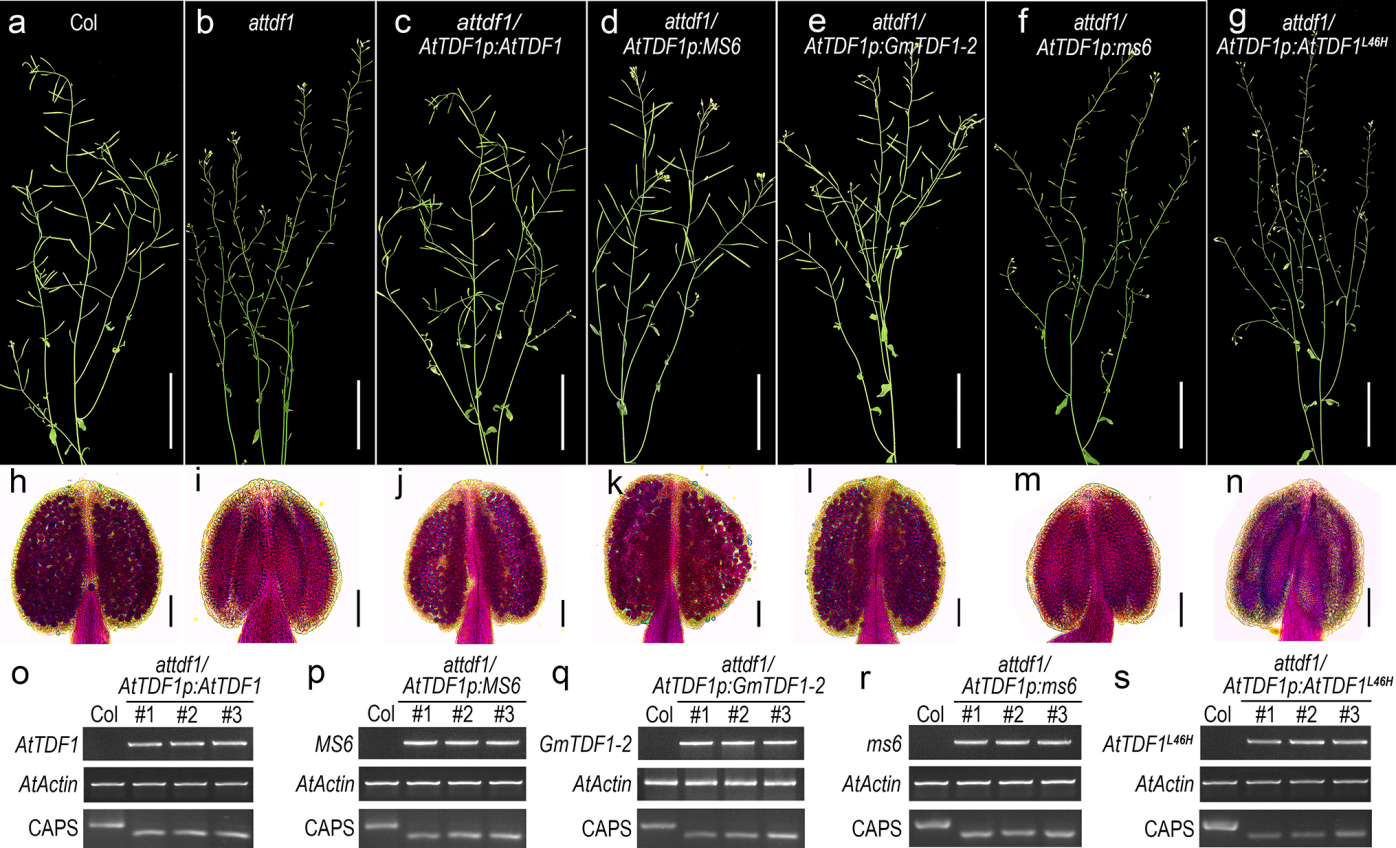
GmTDF1-1 rescues the fertility of *attdf1* **a**–**g** Seed-setting staged col, *attdf1* and transgenic plants in *attdf1* background as labeled in each picture. Scale bar = 5 cm. **h**–**n** Alexander staining of the pollens from col, *attdf1* and the corresponding transgenic plants placed above them. Scale bar = 100 μm. **o**–**s** Verification of the transgenic lines by RT-PCR and CAPS analyses. Top panels, RT-PCR analysis of the transgenes; Middle panels, RT-PCR analysis of *AtActin* as the native control; bottom panels, CAPS analysis showing the transgenic plants in *attdf1* background

As a result, most *attdf1* plants transformed with *AtTDF1* (14/20, rescued/transformants), *MS6* (19/26, rescued/transformants) or *GmTDF1-2* (8/10, rescued/transformants) were fully complemented, producing functional pollens and elongated siliques as wild type did (Fig. 4a–e, h–l, o–q). This demonstrated both GmTDF1-1 and GmTDF1-2 can substitute for AtTDF1 in *Arabidopsis* and they are functional TDF1s. By contrast, *AtTDF1p:ms6* expressing GmTDF1-1^L76H^ that carries L76H mutation (0/25, rescued/ transformants) failed to complement the *attdf1*’s sterility (Fig. 4a, b, f, h, i, m, r). Similarly, expression of *AtTDF1p*-driven *AtTDF1*^*L46H*^, which encodes a mutant form of AtTDF1 (AtTDF1^L46H^) corresponding to GmTDF1-1^L76H^, in *attdf1* displayed the consistent result. All transformants (0/16, rescued/ transformants) developed just like *attdf1* (Fig. 4a, b, g, h, i, n, s). On the other hand, overexpression (OE) of *ms6* driven by 35S promoter in wild-type *Arabidopsis* did not affect anther development. We obtained 15 T1 transgenic OE lines, all producing normal pollens, in which three independent lines (*ms6OE1* ~ *3*) were shown in Fig. S4. These results strongly supported that the substitution of L76H abolishes the function of GmTDF1-1 and leads to the aberrant male development of *ms6* plants. The results also demonstrated that leucine at position 76 in GmTDF1-1 (position 46 in AtTDF1) is crucial for the function of TDF1.

### GmTDF1-1 is the major functional TDF1 in soybean

**Fig. 5:**
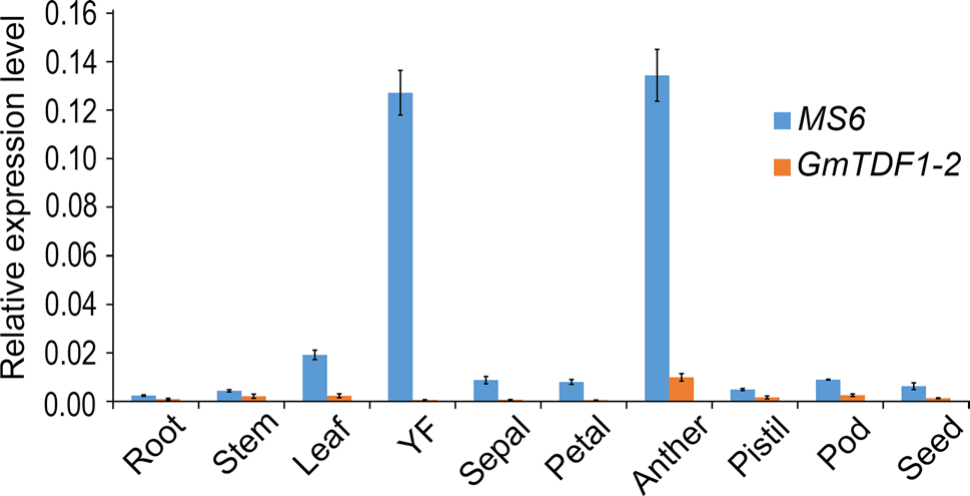
The relative expression patterns of *MS6* and *GmTDF1-2* genes in different tissues The error bars denote ± SD (standard deviation) calculated from three biological replicates, each with three-technique replicates. YF, young flowers (2.5–3.5 mm in length)

Complementary assay above showed that GmTDF1-1 and GmTDF1-2 both were functional TDF1 proteins, which raised the question of why *ms6* exhibits male sterility when there is another TDF1 coding gene in the genome. To answer this question, we characterized the expression levels of *MS6* and *GmTDF1-2* genes by qRT-PCR in roots, stems, leaves, young flowers, pods and immature seeds. *MS6* displayed a tissue-specific expression pattern with a much higher expression level in young flowers (Fig. 5). Then, the expression level of *MS6* in different floral organs were further analyzed, which revealed *MS6* is mainly expressed in anthers, similar to *AtTDF1* and *OsTDF1* (Fig. 5; Zhu et al. 2008; Cai et al. 2015). On the contrary, *GmTDF1-2* is consistently expressed at a low level in all the examined tissues, indicating it is likely in the process of pseudogenization (Fig. 5). Different expression patterns of *MS6* and *GmTDF1-2* illustrated that GmTDF1-1 is the major TDF1 protein functioning in soybean anther development, and therefore the mutation at the *MS6* locus leads to male sterility.

### L76H mutation does not alter the subcellular localization or transactivation activity of GmTDF1-1

**Fig. 6:**
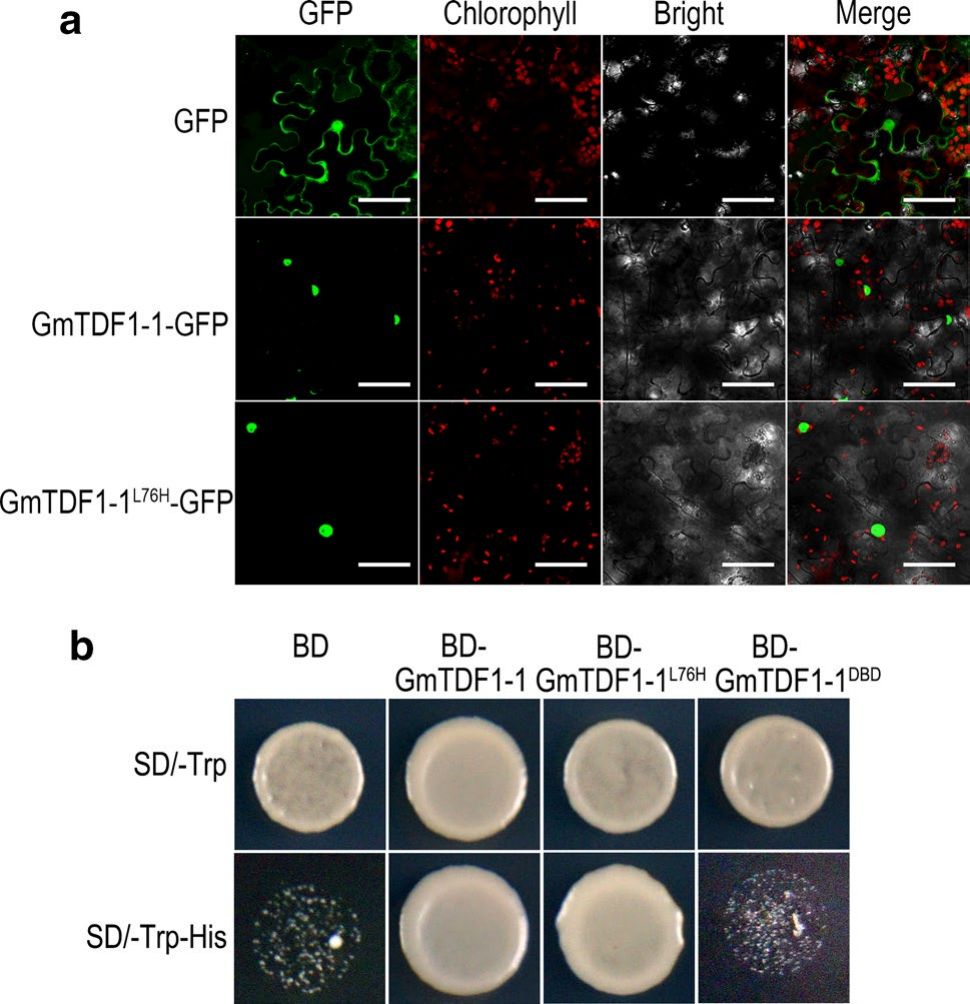
GmTDF1-1 is located in the nucleus and possesses transactivation activity **a** Transient expression of GFP, GmTDF1-1-GFP and GmTDF1-1^L76H^ -GFP in *N. benthamiana* leaves. For each protein, the images of GFP, chloroplast auto-fluorescence, bright field and merged signals are presented. Scale bar = 50 μm. **b** Transactivation analysis of GmTDF1-1, GmTDF1-1^L76H^ and the DNA binding domain of GmTDF1-1 (GmTDF1-1^DBD^) in yeast. BD, the GAL4 DNA binding domain. BD alone was used as the negative control

The subcellular localizations of GmTDF1-1 and GmTDF1-1^L76H^ were analyzed by transiently expressing their GFP fusion proteins driven by *35S* promoter in *N. benthamiana* leaves. Free GFP was also expressed as a control, which showed signals all over the cells (Fig. 6a). Comparatively, the fluorescence of GmTDF1-1-GFP and GmTDF1-1^L76H^-GFP was restricted in the nucleus, in agreement with the general subcellular distribution of transcription factors (Fig. 6a), and the result reveals that the L76H mutation did not affect the subcellular localization of GmTDF1-1.

TDF1 is a transcriptional activator in *Arabidopsis* and rice; therefore, GmTDF1-1 should possess transcription activation activity in soybean as well. We then performed a transactivation activity test in yeast (Fig. 6b). Yeast clones expressing GAL4 DNA binding domain (BD) only grew on the SD medium lack of tryptophan (SD/-Trp) but not on the selective medium (SD/-Trp-His) due to no transactivation activity in the BD region. A similar phenomenon was observed for the yeast clone expressing the BD-fused DNA binding domain of GmTDF1-1 (BD-GmTDF1-1^DBD^). In contrast, yeast clones expressing BD-GmTDF1-1 and BD-GmTDF1-1^L76H^ grew well on both SD/-Trp and SD/-Trp-His mediums, showing the L76H substitution does not influence the transactivation activity of GmTDF1-1. As L76 is a conserved residue in the R2 region of the DNA binding domain (Fig. 2d and f) and L76H has no effect on protein’s subcellular location or transactivation activity, we suspected that the L76H mutation likely disrupted the function of GmTDF1-1 by altering its DNA binding capacity.

### DYT1-TDF1-AMS-MYB80/MYB103/MS188-MS1 pathway downstream TDF1 is severely down regulated in the *ms6* mutant

**Fig. 7:**
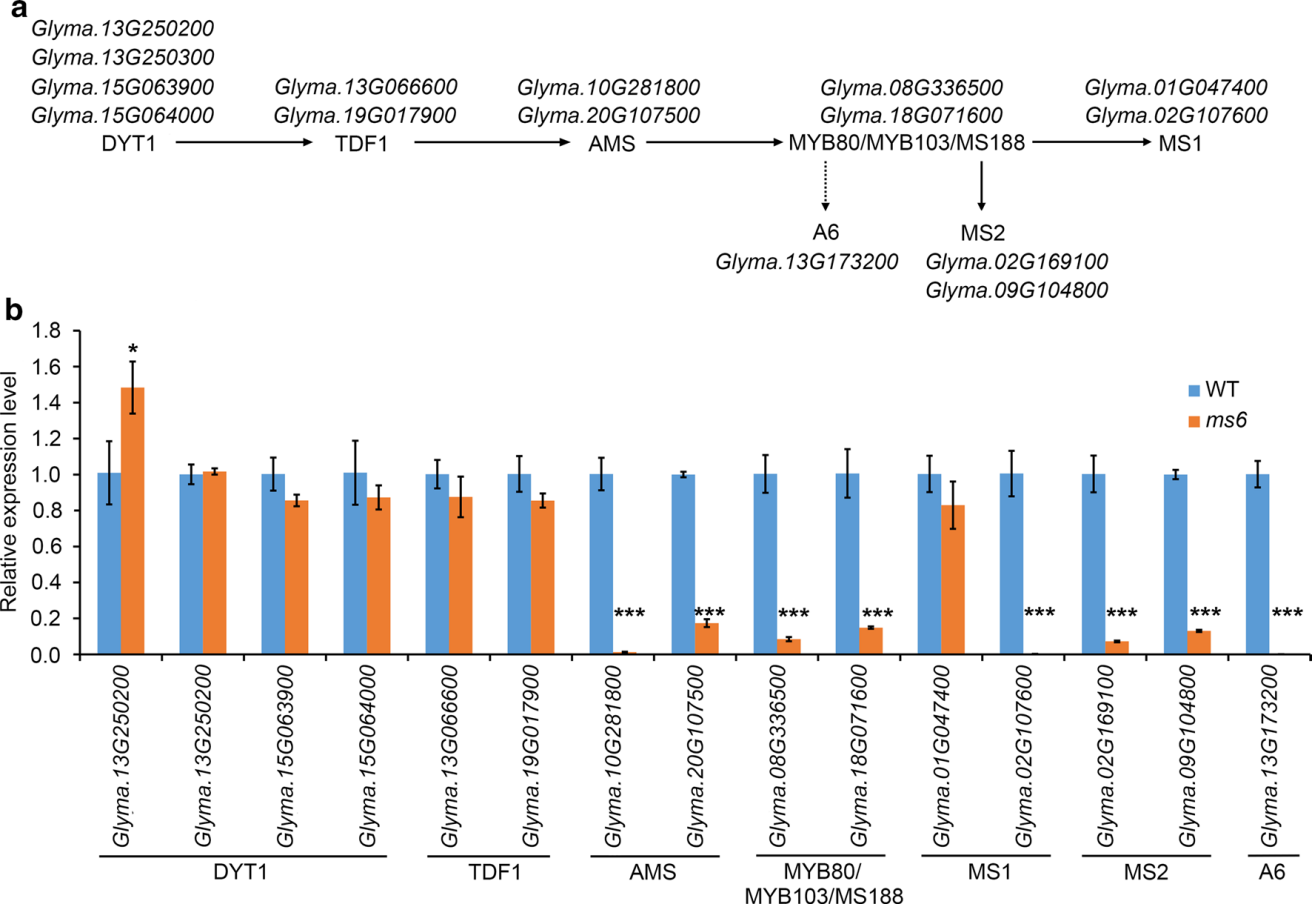
The relative expression levels of anther development core regulators in WT and *ms6* **a** Summarization of soybean genes encoding the TDF1-related regulatory pathway required for anther development. DYT1, TDF1, AMS1, MYB80/MYB103/MS188 and MS1 constitute the ordered transcription factor cascade identified in *Arabidopsis*. The genes encoding MS2 and A6 are well-known target genes of MYB80/MYB103/MS188 in *Arabidopsis*. Solid arrows indicate the direct activation; the dotted arrow indicates the indirect activation. **b** Relative expression levels of the above genes in WT and *ms6* young flowers. The error bars denote ± SD calculated from three biological replicates, each with three-technique replicates. *P* values were calculated by using Student’s *t* test (*P < 0.05, **P < 0.01, ***P < 0.001)

The above experiments showed that GmTDF1-1 is the major functional TDF1 homolog in soybean. In *Arabidopsis*, TDF1 is the critical component in a well-characterized genetic pathway regulating tapetal development and pollen wall formation. This pathway is composed of five transcription factors, which are two basic helix-loop-helix (bHLH) factors (DYSFUNCTIONAL TAPETUM 1 (DYT1) and ABORTED MICROSPORES (AMS)), two MYB factors (TDF1 and MYB80/MYB103/MS188) and one PHD-finger protein (MALE STERILITY 1 (MS1)) (Zhu et al. 2011; Lu et al. 2020). In this pathway, DYT1 directly activates the expression of *TDF1*, and TDF1 subsequently promotes the expression of *AMS*. Then, AMS is required for the expression of the gene encoding MYB80/MYB103/MS188, which is an activator critical for expressing *MS1* (Fig. 7a). Depletion of any member in this cascade would lead to distorted tapetum and aborted pollens (Wilson et al. 2001; Sorensen et al. 2003; Zhang et al. 2006, 2007). The same regulatory cascade known as UDT1-TDF1-TDR-OsMS188-PTC1 was identified in rice as well (Cai et al. 2015).

Soybean *ms6* exhibits similar cytological abnormalities to the null mutants, *attdf1* and *ostdf1*, such as vacuolated tapetum cells, undissolved callose and crushed microspores (Fig. 1f, h; Ilarslan et al. 1999; Zhu et al. 2008; Cai et al. 2015). *AtTDF1p*-driven *MS6* was able to recover the fertility of the *attdf1* mutant as *AtTDF1p*-driven *OsTDF1* did (Fig. 4d, k, p; Cai et al. 2015). These results suggested that TDF1’s function is conserved in *Arabidopsis*, rice and soybean, and implied that GmTDF1-1 controls anther development through the DYT1-TDF1-AMS-MYB80/MYB103/MS188-MS1 regulatory pathway as well. Through the homology search, the other four transcription factors in this pathway were also identified in soybean, in which DYT1 has four paralogs and the others have two paralogs (Fig. 7a). The expression levels of all the genes encoding the components of the pathway in WT and *ms6* young flowers were assessed and compared by qRT-PCR analysis. Among the four genes encoding DYT1 homologs in soybean (GmDYT1s), the expression level of *Glyma.13G250200* showed a significant increase in *ms6* compared to that in WT, consisting to the previous report from *Arabidopsis* that TDF1 negatively feedback-regulates the expression of *DYT1* who encodes the only transcription factor upstream TDF1 in the pathway (Cai et al. 2015). However, the expression levels of the other three genes encoding GmDYT1s were not significantly altered in *ms6* (Fig. 7b), indicating the gene functions of *GmDYT1s* might have diverged. Moreover, the expressions levels of *MS6* and *GmTDF1-2* were not affected in *ms6* (Fig. 7b), suggesting that soybean TDF1, GmTDF1-1, does not regulate the expressions of its own gene and its homolog *GmTDF1-2*.

On the other hand, expressions of the genes encoding the transcription factors downstream of TDF1 in the pathway, like AMS, MYB80/MYB103/MS188 and MS1, were mostly severely down regulated in *ms6* compared to those in WT as expected (Fig. 7b). One exception was *Glyma.01G047400* that encodes one of the MS1 homologs. It expressed similarly in WT and *ms6* (Fig. 7b), implying that *Glyma.01G047400* is functionally diverged from its paralog *Glyma.02G107600* or even might lose the function in anther development. Moreover, we examined the expressions of two structural genes, *A6* and *MALE STERILE 2* (*MS2*), that are regulated by the transcription factor downstream of TDF1 (Zhang et al. 2007; Zhu et al. 2008; Wang et al. 2018). The *A6* gene only has one homolog in soybean genome while *MS2* has two (Fig. 7a), and the expressions of all three genes were strongly suppressed in *ms6* flowers (Fig. 7b). *A6* encodes a *β*-1,3-glucanase required to degrade callose (Hird et al. 1993), which explains why callose surrounding tetrads was not dissolved in *ms6*. *MS2* encodes a fatty acyl reductase catalyzing the palmitoyl-acyl carrier protein into fatty alcohol, a precursor of the major component of pollen wall (Aarts et al. 1997; Chen et al. 2011b; Wang et al. 2018). In *Arabidopsis*, *ms2* mutation disturbs the formation of pollen wall, which results in collapsed microspores and gives rise to no pollen anthers (Aarts et al. 1997). Similarly, no pollen is produced in anthers of soybean *ms6* (Fig. 1d).

Taken together, in soybean *ms6*, the examined well-characterized downstream genes of TDF1 are severely down regulated, while expressions of the genes encoding TDF1 (GmTDF1-1 and GmTDF1-2) and the transcription factor upstream of TDF1 (DYT1) are not suppressed. It indicates that the DYT1-TDF1-AMS-MYB80/MYB103/MS188-MS1 genetic pathway is likely present in soybean and functions conservatively to assure anther development. It also reflects that the DYT1-TDF1-AMS-MYB80/MYB103/MS188-MS1 genetic pathway is blocked at the step regulated by TDF1 in the *ms6* mutant, demonstrating that the mutation of *Glyma.13G066600/MS6* that encodes the major TDF1 in soybean is the determinant for the male sterility of *ms6*.

## Discussion

Plant male sterile mutants are important materials for studying anther development and crucial tools for crop hybrid breeding. So far, 13 genetic loci have been reported to condition the male sterile phenotype in soybean, including *ms1*-*ms9*, *msp, msMOS*, *mst-M* and *ms*_*NJ*_ (Yang et al. 2014; Zhao et al. 2019; Nie et al. 2019; Thu et al. 2019). Nevertheless, only *ms4* has been molecularly identified, which is caused by the mutation of *Glyma.02G243200*, a gene encoding a PHD-finger protein involved in the meiosis process of microsporocyte (Thu et al. 2019). In the present study, we cloned another male sterile gene, *MS6*, by characterizing the *ms6* allele maintained in the germplasm T295H. Results from genetic mapping, comparative high-throughput sequencing, conservation study, phylogenetic analysis, complementary experiment, gene expression patterns and differential expressions of genes in the TDF1-involved regulatory pathway all lead to the conclusion that the male sterility of *ms6* from T295H is caused by the missense mutation at the *Glyma.13G066600* locus (*MS6*), which encodes a soybean homolog of TDF1, GmTDF1-1 (Figs. 2-5, 7). TDF1 is a well-known R2R3 MYB transcription factor required for appropriate tapetum development (Zhu et al. 2008; Cai et al. 2015). The missense mutation in the *ms6* allele results in the substitution of a highly conserved leucine residue by a histidine residue at the position 76 (L76H) in the R2 region of GmTDF1-1 (Fig. 2). The mutation abolishes the function of GmTDF1-1 (Fig. 4), likely by interfering its DNA-binding activity, because the transactivation activity and subcellular distribution of GmTDF1-1^L76H^ encoded by the *ms6* allele are not disturbed (Fig. 6) and the *ms6* allele is expressed at a similar level to that in WT (Fig. 7). Phylogenetic and complementation analyses showed that GmTDF1-1 has a recently diverged and functional paralog, GmTDF1-2 (Figs. 3, 4). However, the *GmTDF1-2* gene is expressed constitutively at a very low level; therefore, it may only have a trivial contribution to the anther development if there is any, and its presence cannot compensate for the deficiency of *MS6* (Fig. 5).

TDF1 is conservatively present in angiosperm species (Fig. 3), regulating the tapetal and microspore development (Zhu et al. 2008; Cai et al. 2015). The major TDF1-involved genetic pathway is the ordered transcription factor cascade identified as DYT1-TDF1-AMS-MYB80/MYB103/MS188-MS1 in *Arabidopsis* (Zhu et al. 2011; Lu et al. 2020) and UDT1-TDF1-TDR-OsMS188-PTC1 in rice (Cai et al. 2015). Expressions of rice and soybean TDF1s (OsTDF1, GmTDF1-1 and GmTDF1-2) in *Arabidopsis attdf1* mutant under the control of the native *AtTDF1* promoter can recover the fertility of the mutant plants, indicating that TDF1’s major functions are quite conserved (Fig. 4; Cai et al. 2015). However, slight divergences of *TDF1* genes are present in different species. For example, in situ hybridization showed that *AtTDF1* was expressed strongly and equivalently in tapetum and meiocytes at stage 6, while *OsTDF1* was expressed much stronger in tapetum than in meiocytes at the similar development stage (Zhu et al. 2008, 2011; Cai et al. 2015). Expressing *OsTDF1* in *attdf1* only partially recovered the expression levels of downstream target genes like *AMS*, *MYB80/MYB103/MS188* and *MS1* (Zhu et al. 2008, 2011; Cai et al. 2015). The function of the major *TDF1* in soybean (*MS6*) is likely more diverged. For instance, mutants *attdf1* and *ostdf1* process meiosis successfully to generate well-separated tetrads, while both soybean *ms6* mutants (Ames1 and Ames2) produce partially- or non-separated multi-nucleic tetrads (Fig. 1f; Skorupska and Palmer 1989; Ilarslan et al. 1999). Additionally, compared to *Arabidopsis* and rice, soybean possesses an extra anther wall layer between the tapetal and middle layers, termed as the parietal layer (Fig. 1; Ilarslan et al. 1999). In *ms6*, the parietal layer is vacuolated and obsessively enlarged, indicating that *MS6* also plays an important role in regulating the parietal layer’s development. To dissect the function of GmTDF1-1 in soybean anther development, future investigation of the GmTDF1-1 downstream network by using RNA-Seq and ChIP-seq technologies is needed.

Stable GMS mutants can facilitate crop improvement via the canonical recurrent selection strategy (Lewers et al. 1996) or the novel GMS-based hybrid breeding system, SPT (Perez-Prat and van Lookeren Campagne 2002; Weber et al. 2009). The main idea of the SPT technology is to create a hemizygous transgenic maintainer line by introducing a resistance gene (for screening transgenic lines) and a special gene cluster (for producing viable *ms* pollens) to a recessive sporophytic *ms* mutant. The gene cluster is composed of at least three fundamental genes (Wu et al. 2016). The first one is the wild-type *MS* gene for rescuing *ms*’s detrimental effects on anther sporophytic cells. The 2nd one is a male gametophyte-killer gene for killing the male gametes carrying the transgenic component, and therefore only non-transgenic *ms* pollens are viable for fertilization and seed production (Chang et al. 2016; Song et al. 2021). The 3rd one is a phenotypic reporter gene for monitoring the purity of the propagated *ms* seeds, such as fluorescence gene expressed in the aleurone layer of seeds (Chang et al. 2016; Zhang et al. 2018b) or anthocyanin synthesis gene expressed in young seedlings (Du et al. 2020). The SPT system broadens the germplasm choices of parental lines to breed hybrids with superior heterosis, reduces the risk caused by weather changes and is regarded as the third generation of hybrid technology (Song et al. 2021).

Therefore, the recessive sporophytic *ms* mutants conditioned by well-characterized *MS* genes are critical for developing the SPT system. However, compared to the major crops like rice and maize with dozens of *ms* mutants controlled by different loci (Guo and Liu 2012; Wan et al. 2019), soybean only has 13 *ms* loci reported (Yang et al. 2014; Zhao et al. 2019; Nie et al. 2019; Thu et al. 2019). One major reason is that soybean is a paleopolyploid with two recent rounds of whole-genome duplication (WGD) occurring ~ 13 and ~ 59 million years ago, and about 75% of the genes exist with multiple copies (Schmutz et al. 2010). For example, among the genes we investigated in the present study, all but *A6* have ≥ 2 paralogs in soybean genome (Fig. 7a). Therefore, there is a big chance that spontaneous mutation at one anther-development-related gene would not affect male gametogenesis due to gene redundancy. In hence, the *ms* mutants are hardly achieved in soybean through traditional random-mutagenesis approaches. Among the reported *ms* mutants, sporophytic *ms6* is an ideal genetic material for the SPT technology due to its stable no pollen phenotype (Fig. 1c, d; Skorupska and Palmer 1989; Ilarslan et al. 1999). Identification of the *MS6* gene in the present study provides an essential element in establishing the potential *ms6*-based SPT system for soybean hybrid-seed production. Elucidation of the mutation site at the *ms6* allele also provides an accurate marker to facilitate soybean improvement via recurrent selection.

## Supplementary Information

Below is the link to the electronic supplementary material.

Supplementary file1 (PDF 810 KB)

## References

[CR1] Aarts MG, Hodge R, Kalantidis K, Florack D, Wilson ZA, Mulligan BJ, Stiekema WJ, Scott R, Pereira A (1997). The *Arabidopsis MALE STERILITY 2* protein shares similarity with reductases in elongation/condensation complexes. Plant J.

[CR2] Bassam BJ, Caetano-Anollés G, Gresshoff PM (1991). Fast and sensitive silver staining of DNA in polyacrylamide gels. Anal Biochem.

[CR3] Cai CF, Zhu J, Lou Y, Guo ZL, Xiong SX, Wang K, Yang ZN (2015). The functional analysis of *OsTDF1* reveals a conserved genetic pathway for tapetal development between rice and *Arabidopsis*. Sci Bull.

[CR4] Chang Z, Chen Z, Wang N, Xie G, Lu J, Yan W, Zhou J, Tang X, Deng XW (2016). Construction of a male sterility system for hybrid rice breeding and seed production using a nuclear male sterility gene. Proc Natl Acad Sci U S A.

[CR5] Chen DC, Yang BC, Kuo TT (1992). One-step transformation of yeast in stationary phase. Curr Genet.

[CR6] Chen L, Lei D, Tang W, Xiao Y (2011). Thoughts and practice on some problems about research and application of two-line hybrid rice. Rice Sci.

[CR7] Chen W, Yu XH, Zhang K, Shi J, Oliveira SD, Schreiber L, Shanklin J, Zhang D (2011). *Male Sterile2* encodes a plastid-localized fatty acyl carrier protein reductase required for pollen exine development in Arabidopsis. Plant Physiol.

[CR8] Clough SJ, Bent AF (1998). Floral dip: a simplified method for *Agrobacterium*-mediated transformation of *Arabidopsis thaliana*. Plant J.

[CR9] Dubos C, Stracke R, Grotewold E, Weisshaar B, Martin C, Lepiniec L (2010). MYB transcription factors in *Arabidopsis*. Trends Plant Sci.

[CR10] Du M, Zhou K, Liu Y, Deng L, Zhang X, Lin L, Zhou M, Zhao W, Wen C, Xing J, Li CB, Li C (2020). A biotechnology-based male-sterility system for hybrid seed production in tomato. Plant J.

[CR11] Guo JX, Liu YG (2012). Molecular control of male reproductive development and pollen fertility in rice. J Integr Plant Biol.

[CR12] Hird DL, Worrall D, Hodge R, Smartt S, Paul W, Scott R (1993). The anther-specific protein encoded by the *Brassica napus* and *Arabidopsis thaliana A6* gene displays similarity to β-1,3-glucanases. Plant J.

[CR13] Ho SN, Hunt HD, Horton RM, Pullen JK, Pease LR (1989). Site-directed mutagenesis by overlap extension using the polymerase chain reaction. Gene.

[CR14] Ilarslan H, Horner HT, Palmer RG (1999). Genetics and cytology of a new male-sterile, female-fertile soybean mutant. Crop Sci.

[CR15] Jin H, Martin C (1999). Multifunctionality and diversity within the plant *MYB*-gene family. Plant Mol Biol.

[CR16] Kim YJ, Zhang D (2018). Molecular control of male fertility for crop hybrid breeding. Trends Plant Sci.

[CR17] Lander ES, Green P, Abrahamson J, Barlow A, Daly MJ, Lincoln SE, Newburg L (1987). MAPMAKER: an interactive computer package for constructing primary genetic linkage maps of experimental and natural populations. Genomics.

[CR18] Levings CS (1990). The Texas cytoplasm of maize: cytoplasmic male sterility and disease susceptibility. Science.

[CR19] Lewers KS, Martin SKS, Widrlechner MP, Palmer RG, Hedges BR (1996). Hybrid soybean seed production: comparison of three methods. Crop Sci.

[CR20] Livak KJ, Schmittgen TD (2001). Analysis of relative gene expression data using real-time quantitative PCR and the 2^-∆∆Ct^ method. Methods.

[CR21] Lu JY, Xiong SX, Yin W, Teng XD, Lou Y, Zhu J, Zhang C, Gu JN, Wilson ZA, Yang ZN (2020). MS1, a direct target of MS188, regulates the expression of key sporophytic pollen coat protein genes in arabidopsis. J Exp Bot.

[CR22] Nie Z, Zhao T, Liu M, Dai J, He T, Lyu D, Zhao J, Yang S, Gai J (2019). Molecular mapping of a novel male-sterile gene *ms*_*NJ*_ in soybean [*Glycine max* (L.) Merr.]. Plant Reprod.

[CR23] Perez-Prat E, van Lookeren Campagne MM (2002). Hybrid seed production and the challenge of propagating male-sterile plants. Trends Plant Sci.

[CR24] Peterson R, Slovin JP, Chen C (2010). A simplified method for differential staining of aborted and non-aborted pollen grains. Int J Plant Biol.

[CR25] Palmer RG, Gai J, Sun H, Burton JW (2001) Production and evaluation of hybrid soybean. In: J. Janick (ed) Plant breeding reviews, Vol 21. John Wiley & Sons, New York, pp 263-307. 10.1002/9780470650196.ch7

[CR26] Palmer RG, Holland JB, Lewers KS (1998). Recombination values for the *Ms6-W1* chromosome region in different genetic backgrounds in soybean. Crop Sci.

[CR27] Sanders PM, Bui AQ, Weterings K, McIntire KN, Hsu YC, Lee PY, Truong MT, Beals TP, Goldberg RB (1999). Anther developmental defects in *Arabidopsis thaliana* male-sterile mutants. Sex Plant Reprod.

[CR28] Schmutz J, Cannon SB, Schlueter J, Ma J, Mitros T, Nelson W, Hyten DL, Song Q, Thelen JJ, Cheng J, Xu D, Hellsten U, May GD, Yu Y, Sakurai T, Umezawa T, Bhattacharyya MK, Sandhu D, Valliyodan B, Lindquist E, Peto M, Grant D, Shu S, Goodstein D, Barry K, Futrell-Griggs M, Abernathy B, Du J, Tian Z, Zhu L, Gill N, Joshi T, Libault M, Sethuraman A, Zhang XC, Shinozaki K, Nguyen HT, Wing RA, Cregan P, Specht J, Grimwood J, Rokhsar D, Stacey G, Shoemaker RC, Jackson SA (2010). Genome sequence of the paleopolyploid soybean. Nature.

[CR29] Skorupska H, Palmer RG (1989). Genetics and cytology of the *ms6* male-sterile soybean. J Hered.

[CR30] Song S, Wang T, Li Y, Hu J, Kan R, Qiu M, Deng Y, Liu P, Zhang L, Dong H, Li C, Yu D, Li X, Yuan D, Yuan L, Li L (2021). A novel strategy for creating a new system of third-generation hybrid rice technology using a cytoplasmic sterility gene and a genic male-sterile gene. Plant Biotechnol J.

[CR31] Sorensen AM, Kröber S, Unte US, Huijser P, Dekker K, Saedler H (2003). The *Arabidopsis ABORTED MICROSPORES* (*AMS*) gene encodes a MYC class transcription factor. Plant J.

[CR32] Takagi H, Abe A, Yoshida K, Kosugi S, Natsume S, Mitsuoka C, Uemura A, Utsushi H, Tamiru M, Takuno S, Innan H, Cano LM, Kamoun S, Terauchi R (2013). QTL-seq: rapid mapping of quantitative trait loci in rice by whole genome resequencing of DNA from two bulked populations. Plant J.

[CR33] Tamura K, Stecher G, Peterson D, Filipski A, Kumar S (2013). MEGA6: molecular evolutionary genetics analysis version 6.0. Mol Biol Evol.

[CR34] Thu SW, Rai KM, Sandhu D, Rajangam A, Balasubramanian VK, Palmer RG, Mendu V (2019). Mutation in a PHD-finger protein MS4 causes male sterility in soybean. BMC Plant Biol.

[CR35] Wang K, Guo ZL, Zhou WT, Zhang C, Zhang ZY, Lou Y, Xiong SX, Yao XZ, Fan JJ, Zhu J, Yang ZN (2018). The regulation of sporopollenin biosynthesis genes for rapid pollen wall formation. Plant Physiol.

[CR36] Wan X, Wu S, Li Z, Dong Z, An X, Ma B, Tian Y, Li J (2019). Maize genic male-sterility genes and their applications in hybrid breeding: progress and perspectives. Mol Plant.

[CR37] Weber N, Commuri P, Rood T, Townsend R (2009) Petition for the determination of nonregulated status for Maize 32138 SPT maintainer used in the Pioneer Seed Production Technology (SPT) process. Submitted to the USDA-APHIS by Pioneer Hi-Bred International, Inc. Available at: http:// www.aphis.usda.gov/brs/aphisdocs/08_33801p.pdf (last accessed 13 August 2015)

[CR38] Wilson ZA, Morroll SM, Dawson J, Swarup R, Tighe PJ (2001). The *Arabidopsis MALE STERILITY 1* (*MS1*) gene is a transcriptional regulator of male gametogenesis, with homology to the PHD-finger family of transcription factors. Plant J.

[CR39] Wu Y, Fox TW, Trimnell MR, Wang L, Xu RJ, Cigan AM, Huffman GA, Garnaat CW, Hershey H, Albertsen MC (2016). Development of a novel recessive genetic male sterility system for hybrid seed production in maize and other cross-pollinating crops. Plant Biotechnol J.

[CR40] Yang Y, Speth BD, Boonyoo N, Baumert E, Atkinson TR, Palmer RG, Sandhu D (2014). Molecular mapping of three male-sterile, female-fertile mutants and generation of a comprehensive map of all known male sterility genes in soybean. Genome.

[CR41] Zhang D, Luo X, Zhu L (2011). Cytological analysis and genetic control of rice anther development. J Genet Genomics.

[CR42] Zhang D, Chang E, Yu X, Chen Y, Yang Q, Cao Y, Li X, Wang Y, Fu A, Xu M (2018). Molecular characterization of magnesium chelatase in Soybean [Glycine max (L.) Merr.]. Front Plant Sci.

[CR43] Zhang D, Wu S, An X, Xie K, Dong Z, Zhou Y, Xu L, Fang W, Liu S, Liu S, Zhu T, Li J, Rao L, Zhao J, Wan X (2018). Construction of a multicontrol sterility system for a maize male-sterile line and hybrid seed production based on the *ZmMs7* gene encoding a PHD-finger transcription factor. Plant Biotechnol J.

[CR44] Zhang W, Sun Y, Timofejeva L, Chen C, Grossniklaus U, Ma H (2006). Regulation of *Arabidopsis* tapetum development and function by *DYSFUNCTIONAL TAPETUM1* (*DYT1*) encoding a putative bHLH transcription factor. Development.

[CR45] Zhang ZB, Zhu J, Gao JF, Wang C, Li H, Li H, Zhang HQ, Zhang S, Wang DM, Wang QX, Huang H, Xia HJ, Yang ZN (2007). Transcription factor *AtMYB103* is required for anther development by regulating tapetum development, callose dissolution and exine formation in Arabidopsis. Plant J.

[CR46] Zhao L, Sun H, Wang S, Wang Y, Huang M, Li J (2004). Breeding of hybrid soybean HybSoy1. Chinese J Oil Crop Sci.

[CR47] Zhao Q, Tong Y, Yang C, Yang Y, Zhang M (2019). Identification and mapping of a new soybean male-sterile gene, *mst-M*. Front Plant Sci.

[CR48] Zhu J, Chen H, Li H, Gao JF, Jiang H, Wang C, Guan YF, Yang Z (2008). Defective in *Tapetal Development and Function 1* is essential for anther development and tapetal function for microspore maturation in arabidopsis. Plant J.

[CR49] Zhu J, Lou Y, Xu X, Yang ZN (2011). A genetic pathway for tapetum development and function in *arabidopsis*. J Integr Plant Biol.

