# Supplementary material for: A single nucleotide polymorphism in an R2R3 MYB transcription factor gene triggers the male sterility in soybean ms6 (Ames1)
Source: Theor Appl Genet. 2021 Jul 28;134(11):3661–74. doi: 10.1007/s00122-021-03920-0 (PMC8519818; doi:10.1007/s00122-021-03920-0)
Supplement: Supplementary file 1 — Supplementary file1 (PDF 810 KB) [file 122_2021_3920_MOESM1_ESM.pdf]

Theoretical and Applied Genetics

# A single nucleotide polymorphism in an R2R3 MYB transcription factor gene triggers the male sterility in soybean *ms6* (Ames1)

Junping Yu[1#], Guolong Zhao[2#], Wei Li[1#], Ying Zhang[2], Peng Wang[1], Aigen Fu[1], Limei Zhao[2], Chunbao Zhang[2]*, Min Xu[1]*

1 Chinese Education Ministry's Key Laboratory of Western Resources and Modern Biotechnology, Key Laboratory of Biotechnology Shaanxi Province, College of Life Sciences, Northwest University, Xi'an, 710069, China

2 Soybean Research Institute, National Engineering Research Center for Soybean, Jilin Academy of Agricultural Sciences, Changchun, 130033, China

[#]These authors contribute equally to this work.

***Corresponding emails: Min Xu**, minxu@nwu.edu.cn; **Chunbao Zhang**, cbzhang@cjaas.com

**ORCID IDs: Min Xu**, 0000-0002-7206-0059; **Chunbao Zhang**, 0000-0001-9344-9162

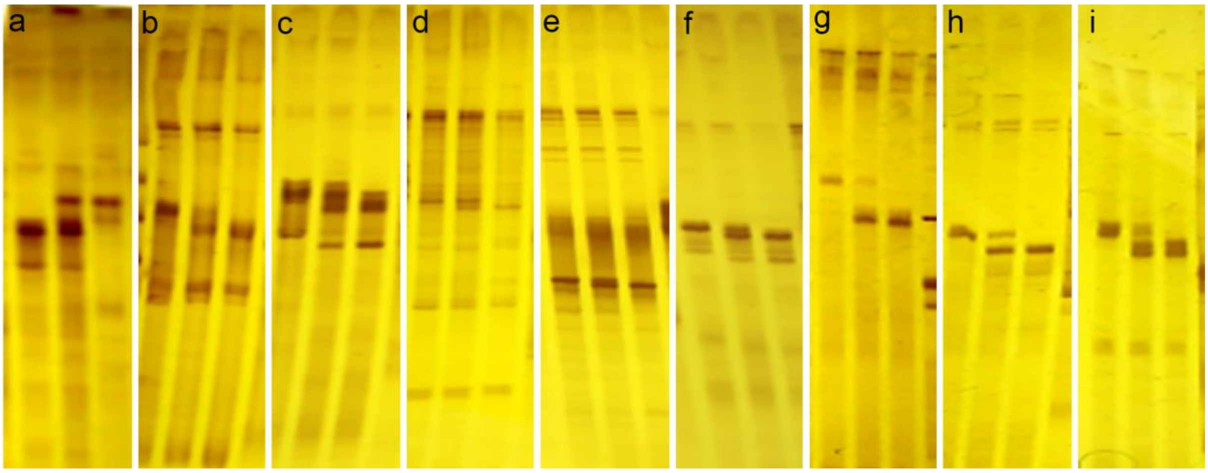


**Fig. S1** The PAGE patterns of SSR markers used in the fine mapping experiment

For each panel, lane1, ‘JiuB’; lane2, $BC_5F_1$; lane3, *ms6* from T295H. **a** BARCSOYSSR-13-0243; **b** BARCSOYSSR-13-0244; **c** BARCSOYSSR-13-0245; **d** BARCSOYSSR-13-0249; **e** BARCSOYSSR-13-0257; **f** BARCSOYSSR-13-0259; **g** BARCSOYSSR-13-0275; **h** BARCSOYSSR-13-0277; **i** BARCSOYSSR-13-0283.

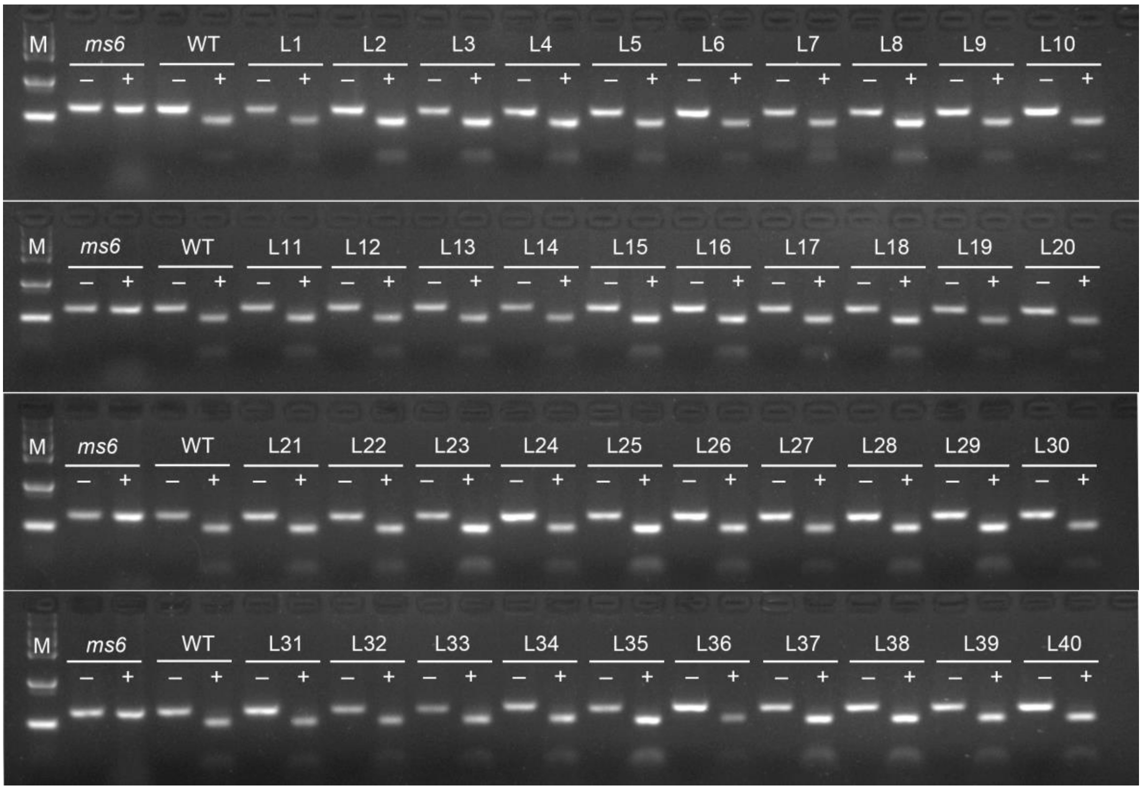


**Fig. S2** CAPS analysis of 40 male fertile soybean accessions with different geographic origins

All 40 accessions (L1 to L40, Table S2) exhibited the WT pattern, showing the CAPS site is well conserved in soybean. -/+, sample without/ with *Mse*I digestion. M, marker.

```
                         *        20         *        40         *        60         *
GmTDF1-1 : MHIASPLHNSLSPSLAFLLLLCLISSLISKMGRPPCCDKSNVKRGLWTPEEDAKILAYVANHGTGNWTLVPKKAGL :  76
GmTDF1-2 : ------------------------------MGRPPCCDKSNVKRGLWTPEEDAKILAYVVNHGTGNWTLVPKKAGL :  46
AtTDF1    : ------------------------------MGRPPCCDKSNVKKGLWTEEEDAKILAYVAIHGVGNWSLIPKKAGL :  46
OsTDF1    : ------------------------------MGRPPCCDKANVKKGPWTAEEDAKLLAYTSTHGTGNWTSVPQRAGL :  46

             80         *       100         *       120         *       140         *
GmTDF1-1 : NRCGKSCRLRWTNYLRPDLKHDGFTPQEEDLIINLHGAIGSRWSLIAKRLPGRTDNDVKNYWNTKLRKKLMKMGID : 152
GmTDF1-2 : NRCGKSCRLRWTNYLRPDLKHDGFTPQEEELIINLHGAIGSRWSIIAKRLPGRTDNDVKNYWNTKLRKKLMKMGID : 122
AtTDF1    : NRCGKSCRLRWTNYLRPDLKHDSFSTQEEELIIECHRAIGSRWSSIARKLPGRTDNDVKNHWNTKLKKKLMKMGID : 122
OsTDF1    : KRCGKSCRLRYTNYLRPNLKHENFTQEEEELIVTLHAMLGSRWSLIANQLPGRTDNDVKNYWNTKLSKKLRQRGID : 122

                160         *       180         *       200         *       220
GmTDF1-1 : PVTHKPVSQVLSDLGSISGLPN-TTNQMAFINKDLMMSNMPP-TKTEPSDSNKSMVEHTQEGQVHSWEHHIPYQVI : 226
GmTDF1-2 : PVTHKPVSQVLSDLGSISGLPNTTTNQIAFINKDLMMSNMLPITKTEPSGSNKSTVEHTQEGQVPSWEHHIPYQVI : 198
AtTDF1    : PVTHKPVSQLLAEFRNISGHGNASFKTEPSNNSILTQSNSAWEMMRNTTTNHESYYTNSPMMFTNSSEYQTTPFHF : 198
OsTDF1    : PITHRPIADLMQSIGTLAIRPPPAAGAAPP-PCLPVFHDAPYFAALQHQHQQQQVVTHVDADAPASPDSQHLQLNW : 197

            *       240         *       260         *       280         *       300
GmTDF1-1 : INSENVQPQVLSEAASSTSSSSSSNLTQLGSPQSYSCQTPQAQISPPCSSFDWSEFLHSDSFNWSLNPPSGLMQSE : 302
GmTDF1-2 : INSENIQSHVLSEAASSTSSSSSSNITQLGSPQSYSCQTPQAQIAPPCSSFDWSEFLQSDSFNWSLNPSSGIIQSE : 274
AtTDF1    : YSHPN---HLLNGTTSSCSSSSSS--TSITQPN----QVPQT----PVTNFYWSDFLLSD-------PVPQVVGSS : 254
OsTDF1    : S-------DFLADDAAGHGADAPAPQAALGQYQ--EGSAP---------------------------AATAVVGGG : 237

                 *       320         *       340         *       360         *
GmTDF1-1 : AELSDNTKSNGHDMQGAASEGSGSGSGAVACGASMEYQINKQCEAHSFVDGILDRDSEIRAAFPQLLDASFDY-- : 375
GmTDF1-2 : AELSNNAKSNGNDMQGGASEGSGS--GAVACGASMEYQINKQCEAHSFVDGILDRDSEIRAAFPELLDASFDC-- : 345
AtTDF1    : ATSDLTFTQNEHHFNIEAEYISQN----IDSKASGTCHS-----ASSFVDEILDKDQEMLSQFPQLLN-DFDY-- : 317
OsTDF1    : RAFG-DVDGASAGVGAGTDDGAGAASAFIDAILDCDKEMG----VDQLIAEMLA-DPAYYGGGGGSSSSELGWGC : 306
```

**Fig. S3** Multiple sequence alignment of GmTDF1-1, GmTDF1-2, AtTDF1 and OsTDF1

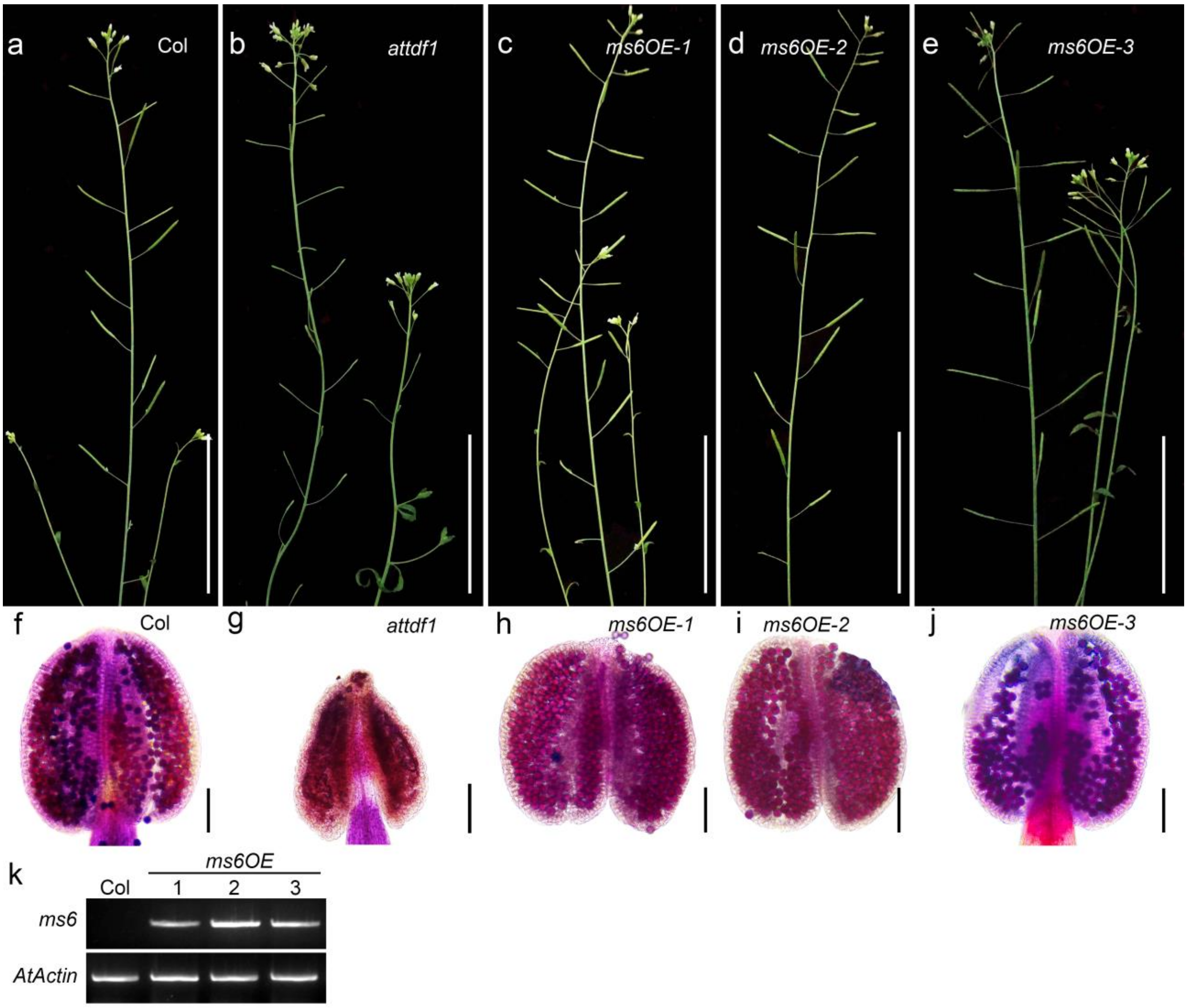


**Fig. S4** Overexpression of *ms6* in *Arabidopsis*

**a-e** Seed-setting staged col, *attdf1* and three independent *ms6* overexpression transgenic plants in Col background (*ms6OE-1~ms6OE-3*) as labeled in each picture. Scale bar = 5 cm. **f-j** Alexander staining of the anther collected from Col, *attdf1* and *ms6OE-1~OE-3*. Scale bar = 100 μm. **k** Accessing the expression level of *ms6* in transgenic plants *ms6OE-1~ ms6OE-3* by RT-PCR. *AtActin* was used as the reference gene.

**Table S1** The list of primers used in this paper

| **Primer Name** | **Sequence (5'-3')** | **Function** |
|---|---|---|
| 13-0243-F | CGTTTCGTGCTAACAAAAATTG | Fine mapping |
| 13-0243-R | AAAATTATTTGTCATCTCATTCATACA | |
| 13-0244-F | TCTCTCCCTTCCACACTCTCA | Fine mapping |
| 13-0244-R | CGAGAGTTAACCTCAAGATTATGC | |
| 13-0245-F | TTGCACATTCTTTTTGGTAAACAGTCATAA | Fine mapping |
| 13-0245-R | GTTGGAGGCCATAGTCACATTAATCTTAGA | |
| 13-0249-F | CAAAGCAAAAGCATGACGAG | Fine mapping |
| 13-0249-R | TCCCACTTAGGATCTTGGTTC | |
| 13-0257-F | CCGTATCCTACCCGTTAATGA | Fine mapping |
| 13-0257-R | CATCGCAAACGAATTGGTAG | |
| 13-0259-F | TTTGGTTATTAGTTATTTTGGCATGA | Fine mapping |
| 13-0259-R | CCACTACTCTCATTATTTTTAAACCG | |
| 13-0275-F | TTGCATATTCAATTATATTTGTTGG | Fine mapping |
| 13-0275-R | CGAACGCGAAGCTAGAAGAT | |
| 13-0277-F | TTTTCTTTCCACCAAAATTTGTA | Fine mapping |
| 13-0277-R | CGCATCATATTAATAAAAAGGATAACA | |
| 13-0283-F | CGATGGCAATTATGCGGTTA | Fine mapping |
| 13-0283-R | TCGTACCTTTCATTTTATCTCGC | |
| MS6CAPS-F | GGCTCTTTTCTTGAACTTGTTTGC | *ms6* genotyping |
| MS6CAPS-R | CCAATTAGATCTTCTTCTTGGGG | |
| AC1F | ATGGGAAGACCTCCTTGTTG | Site direct mutagenesis to create *AtTDF1*$^{L46H}$ |
| AC1R | GCTCTTTCCACATCGATTGT | |
| AC2F | ACAATCGATGTGGAAAGAGC | |
| AC2R | CAATAGGGAAAAGAAGGGATC | |
| KpnI-AtTDF1proF | AAGGTACCCTGTTCTTCAATCTTCACC | pCAMBIA1301-*AtTDF1p* |
| XbaI-AtTDF1proR | TCTAGATTTTTGGTATTCAAACACAAG | |
| MS6F | CCTCTAGAATGCATATTGCATCACCATT | pCAMBIA1301-*AtTDF1p-MS6* |
| BstEII-MS6R | CCGGTCACCTTAGTAGTCAAAAGAAGCATCC | pCAMBIA1301-*AtTDF1p-ms6* |
| AtTDF1F | CCTCTAGAATGGGAAGACCTCCTTGTTG | pCAMBIA1301-*AtTDF1p-AtTDF1* |
| BstEII-AtTDF1R | CCGGTCACCCTAATAATCGAAATCATTCAAGAG | pCAMBIA1301-*AtTDF1p-attdf1*$^{L46H}$ |
| MS6HF | CCTCTAGAATGGGAAGGCCTCCTTGTTG | pCAMBIA1301-*AtTDF1p-GmTDF1-2* |
| BstEII-MS6HR | CCGGTCACCTTAGCAGTCAAAAGAAGCATCC | |
| AtActin-F | CTCTCCCGCTATGTATGTCGCCATCC | *AtActin* RT-PCR |
| AtActin-R | CTGTGAACGATTCCTGGACCTGCCTC | |
| CAPSAtTDF1-F | GCTTGTAACCTCTGATGTTTTC | *attdf1* genotyping |
| CAPSAtTDF1-R | GAGTATAGAGTTGTTAGATGG | |
| NdeI-BK-MS6-F | TCCATATGCATATTGCATCACCATTAC | pGBKT7-GmTDF1-1 |
| EcoRI-BK-MS6-R | GGAATTCTTAGTAGTCAAAAGAAGCATCC | pGBKT7-GmTDF1-1$^{L76H}$ |

| | | |
|---|---|---|
| NdeI-BK-MS6-F | TCCATATGCATATTGCATCACCATTAC | pGBKT7-GmTDF1-1$^{DBD}$ |
| EcoRI-BK-MS6DBD-R | GGAATTCCATGTTGCTCATCATCAAGTC | |
| XbaI-MS6-F | CCTCTAGAATGCATATTGCATCACCATT | pLM-35S-GmTDF1-1-GFP<br>pLM-35S-GmTDF1-1$^{L76H}$-GFP |
| XbaI-MS6-R | CCTCTAGAGTAGTCAAAAGAAGCATCCA | |
| MS6qRT-F | CTCCCCAAGAAGAAGATCTA | *MS6* qRT-PCR |
| MS6qRT-R | GCTTATGTGTCACTGGATCG | |
| MS6HqRT-F | CTCCCCAAGAAGAAGAGCTC | *GmTDF1-2* qRT-PCR |
| MS6HqRT-R | TACCGGCTTATGTGTTACCG | |
| Actin11qRT-F | CGGTGGTTCTATCTTGGCATC | *GmActin11* qRT-PCR |
| Actin11qRT-R | GTCTTTCGCTTCAATAACCCTA | |
| A6qRT-F | TAACAACAATGAAAGCTGGTCG | *Glyma.13G173200* qRT-PCR |
| A6qRT-R | CTGAATTGTAGCTCAGCACTTC | |
| 10g281800qRT-F | TTCCATCCATCAGAACATGTCA | *Glyma.10G281800* qRT-PCR |
| 10g281800qRT-R | CATGAGGCTTTTCTGATACGTG | |
| 20g107500qRT-F | GCAACTGCATGAATTGTGTAAG | *Glyma.20G107500* qRT-PCR |
| 20g107500qRT-R | GTGTTCAATGCCTCCATCAG | |
| 1g047400qRT-1F | AATGAGACATGGTTTGGCAG | *Glyma.01G047400* qRT-PCR |
| 1g047400qRT-1R | TTAGTGGAATGCCATGGCTA | |
| 2g107600qRT-F | GAAAGTTGACGTAGTTGGGAAC | *Glyma.02G107600* qRT-PCR |
| 2g107600qRT-R | GATTCAATGCTGCCTCTATTGG | |
| 2g169100qRT-F | GAGAGGGGATATTCCAGTTG | *Glyma.02G169100* qRT-PCR |
| 2g169100qRT-R | GGGACCACATCTAGTACTCC | |
| 9g104800qRT-F | GCATGTCTGAAGAAGAAAAG | *Glyma.09G104800* qRT-PCR |
| 9g104800qRT-R | ACTGACTCCCCATTCCTCTC | |
| 13g250200qRT-1F | GCAAGCCTTTTGATGCTGCG | *Glyma.13G250200* qRT-PCR |
| 13g250200qRT-1R | GCTCCTTTTGTGGTTGCGAG | |
| 13g250300qRT-F | CGTACCTGAACTAACTTGGGAC | *Glyma.13G250300* qRT-PCR |
| 13g250300qRT-R | ATGGGTCTTGAGGAAAGGAG | |
| 15g063900qRT-F | TTTGATGCTGCGGTCTATGATG | *Glyma.15G063900* qRT-PCR |
| 15g063900qRT-R | CAGAGTTTATTCTCGTTTATTTGCG | |
| 15g064000qRT-F | CCTCTCAAAAAGGTGCACCTT | *Glyma.15G064000* qRT-PCR |
| 15g064000qRT-R | GTGAAAATATTATGAGCTGACACC | |
| 8g336500qRT-F | AATTAACTTCCACCAGGGACAG | *Glyma.08G336500* qRT-PCR |
| 8g336500qRT-R | GGGAAAACACCGTATGGCATTG | |
| 18g071600qRT-F | ATTAACTTCCACCAGGGGCAA | *Glyma.18G071600* qRT-PCR |
| 18g071600qRT-R | GGAAAAACACCGTATGGCATCA | |

**Table S2** Detailed information of 40 accessions used for CAPS analysis to access the conservation of the site mutated in *ms6* allele

| Number | Accession | Improvement level | Origin | Latitude |
|---|---|---|---|---|
| L1 | PI 548203 | Improved cultivars | United States, Iowa | 40°N-43°N |
| L2 | PI 632963 | Improved cultivars | United States, Iowa | 40°N-43°N |
| L3 | PI 632964 | Improved cultivars | United States, Iowa | 40°N-43°N |
| L4 | PI 594895 | Improved cultivars | United States, Iowa | 40°N-43°N |
| L5 | PI 548512 | Improved cultivars | United States, Indiana | 37°N-41°N |
| L6 | Williams 82 | Improved cultivars | United States, Illinois | 36°N-42°'N |
| L7 | Essex | Improved cultivars | United States, Virginia | 36°N-39°'N |
| L8 | Zhonghuang13 | Improved cultivars | China, Bei Jing | 39°N-41°N |
| L9 | Zhonghuang30 | Improved cultivars | China, Bei Jing | 39°N-41°N |
| L10 | Fen Dou 78 | Improved cultivars | China, Shan Xi | 34°N-40°N |
| L11 | XD0632 | Improved cultivars | China, Shaan Xi | 31°N-39°N |
| L12 | Shang Ning 18 | Improved cultivars | China, Shan Dong | 34°N-38°N |
| L13 | He Dou 29 | Improved cultivars | China, An Hui | 29°N-34°N |
| L14 | An 5246 | Improved cultivars | China, An Hui | 29°N-34°N |
| L15 | Fu Bao | Improved cultivars | China, An Hui | 29°N-34°N |
| L16 | FuHui1 | Improved cultivars | China, An Hui | 29°N-34°N |
| L17 | FuHui2 | Improved cultivars | China, An Hui | 29°N-34°N |
| L18 | FuHui9 | Improved cultivars | China, An Hui | 29°N-34°N |
| L19 | M0901 | Improved cultivars | China, An Hui | 29°N-34°N |
| L20 | W931B | Improved cultivars | China, An Hui | 29°N-34°N |
| L21 | WR016 | Improved cultivars | China, An Hui | 29°N-34°N |
| L22 | Jia Lv | Landraces | China, He Nan | 31°N-36°N |
| L23 | Lin You Hei Dou | Landraces | China, Shaan Xi | 31°N-39°N |
| L24 | Yong Shou Hei Dou | Landraces | China, Shaan Xi | 31°N-39°N |
| L25 | Ji Wei 19 | Landraces | China, Hu Nan | 24°N-30°N |
| L26 | YS068 | Wild soybean (*G.soja*) | China, Hei Long Jiang | 43°N-53°N |
| L27 | YS038 | Wild soybean (*G.soja*) | China, Hei Long Jiang | 43°N-53°N |
| L28 | LJ047 | Wild soybean (*G.soja*) | China, Ji Lin | 40°N-46°N |
| L29 | DA041 | Wild soybean (*G.soja*) | China, Ji Lin | 40°N-46°N |
| L30 | LJ049 | Wild soybean (*G.soja*) | China, Ji Lin | 40°N-46°N |
| L31 | DD032 | Wild soybean (*G.soja*) | China, Liao Ning | 38°N-43°N |
| L32 | LNPJ017 | Wild soybean (*G.soja*) | China, Liao Ning | 38°N-43°N |
| L33 | SDHZ14 | Wild soybean (*G.soja*) | China, Shan Dong | 34°N-38°N |
| L34 | SXYP019 | Wild soybean (*G.soja*) | China, Shaan Xi | 31°N-39°N |
| L35 | SXZP061 | Wild soybean (*G.soja*) | China, Shaan Xi | 31°N-39°N |
| L36 | HNXC019 | Wild soybean (*G.soja*) | China, He Nan | 31°N-36°N |
| L37 | HNXC004 | Wild soybean (*G.soja*) | China, He Nan | 31°N-36°N |
| L38 | CQKX003 | Wild soybean (*G.soja*) | China, Chong Qing | 28°N-32°N |
| L39 | CQKX057 | Wild soybean (*G.soja*) | China, Chong Qing | 28°N-32°N |
| L40 | CQYY042 | Wild soybean (*G.soja*) | China, Chong Qing | 28°N-32°N |
